# Healthcare exposures and associated risk of endocarditis after open-heart cardiac valve surgery

**DOI:** 10.1186/s12916-024-03279-1

**Published:** 2024-02-08

**Authors:** Timothy N. Kwan, David Brieger, Vincent Chow, Arnold Chin Tse Ng, Gemma Kwan, Karice Hyun, Raymond Sy, Leonard Kritharides, Austin Chin Chwan Ng

**Affiliations:** 1 Department of Cardiology, Concord Hospital, The University of Sydney, 1 Hospital Road, Concord, NSW 2139 Australia; 2 Department of Cardiology, Princess Alexandra Hospital, Woolloongabba, QLD Australia

**Keywords:** Endocarditis, Cardiac surgery, Epidemiology, Nosocomial infections

## Abstract

**Background:**

Infective endocarditis (IE) following cardiac valve surgery is associated with high morbidity and mortality. Data on the impact of iatrogenic healthcare exposures on this risk are sparse. This study aimed to investigate risk factors including healthcare exposures for post open-heart cardiac valve surgery endocarditis (PVE).

**Methods:**

In this population-linkage cohort study, 23,720 patients who had their first cardiac valve surgery between 2001 and 2017 were identified from an Australian state-wide hospital-admission database and followed-up to 31 December 2018. Risk factors for PVE were identified from multivariable Cox regression analysis and verified using a case-crossover design sensitivity analysis.

**Results:**

In 23,720 study participants (median age 73, 63% male), the cumulative incidence of PVE 15 years after cardiac valve surgery was 7.8% (95% CI 7.3–8.3%). Thirty-seven percent of PVE was healthcare-associated, which included red cell transfusions (16% of healthcare exposures) and coronary angiograms (7%). The risk of PVE was elevated for 90 days after red cell transfusion (HR = 3.4, 95% CI 2.1–5.4), coronary angiogram (HR = 4.0, 95% CI 2.3–7.0), and healthcare exposures in general (HR = 4.0, 95% CI 3.3–4.8) (all *p* < 0.001). Sensitivity analysis confirmed red cell transfusion (odds ratio [OR] = 3.9, 95% CI 1.8–8.1) and coronary angiogram (OR = 2.6, 95% CI 1.5–4.6) (both *p* < 0.001) were associated with PVE. Six-month mortality after PVE was 24% and was higher for healthcare-associated PVE than for non-healthcare-associated PVE (HR = 1.3, 95% CI 1.1–1.5, *p* = 0.002).

**Conclusions:**

The risk of PVE is significantly higher for 90 days after healthcare exposures and associated with high mortality.

**Supplementary Information:**

The online version contains supplementary material available at 10.1186/s12916-024-03279-1.

## Background

Infective endocarditis (IE) is a serious complication after cardiac valve surgery with high morbidity and mortality. Although IE is rare with an incidence of 4–15 cases per 100,000 annually across American and European populations [1–4], the incidence of IE at 10 years after cardiac valve surgery is 1 in 20 [5, 6] (i.e., 20- to 70-fold higher risk) [4, 5]. Some observational studies have reported an increasing incidence of IE [1, 7]. There has been a guideline driven decline in IE prophylaxis prior to invasive procedures [8–10]. This change to guidelines is in the context of a global trend of increasing health care provision to an aging population and rising rates of hospital acquired infections [11]. Hospital exposures may represent a substantial contribution to the rising incidence of IE. Indeed, a statistical relationship between many procedures and IE has been established [12–14].

A more granular understanding of the types of healthcare exposures and the time window at which IE occurs after them is essential to determining who is at risk and who may benefit from prophylaxis. This is particularly relevant for patients with prosthetic valves in whom the risk of post procedural infection is the highest. To the authors’ knowledge, there have only been two large studies evaluating the effect of non-dental healthcare exposures on IE risk in the general population [13, 15] and one large study evaluating the effect of dental exposures [14], with many others limited by small sample size and cross-sectional design. No large longitudinal studies have specifically investigated the epidemiology of post cardiac valve surgery endocarditis (PVE). There is a need for studies to evaluate the effect of healthcare exposures on IE in patients after cardiac valve surgery, especially in the more recent period after changes to antibiotic guidelines (in 2008) [8–10].

We investigated the epidemiology and associations of PVE over 17 years in a large cohort study of Australian patients. We aimed specifically to evaluate healthcare-related exposures as transient risk factors and prognostic factors for PVE.

## Methods

A population-wide linkage study using the New South Wales (NSW) Admitted Patient Data Collection (APDC) database was performed to identify patients who experienced IE requiring hospitalization after a surgical cardiac valve operation between 2001 and 2018 [16]. The APDC database contains ≥ 97% of NSW healthcare facility admission data since 2001 and is part of the Centre for Health Record Linkage (CHeReL) key databases. The CHeReL holds one of the largest data linkage systems in Australia with linked health data on the NSW population. Mortality data were tracked from the NSW Death Registry over the same time period.

The study protocol conforms to the ethical guidelines of the 1975 Declaration of Helsinki. Approval was granted by the NSW Population and Health Services Research Ethics Committee, reference number: 2013/09/479. The ethics committees granted a waiver of the usual requirement for the consent of the individual to the use of their health information. All patient data were deidentified and analyzed anonymously.

**Fig. 1:**
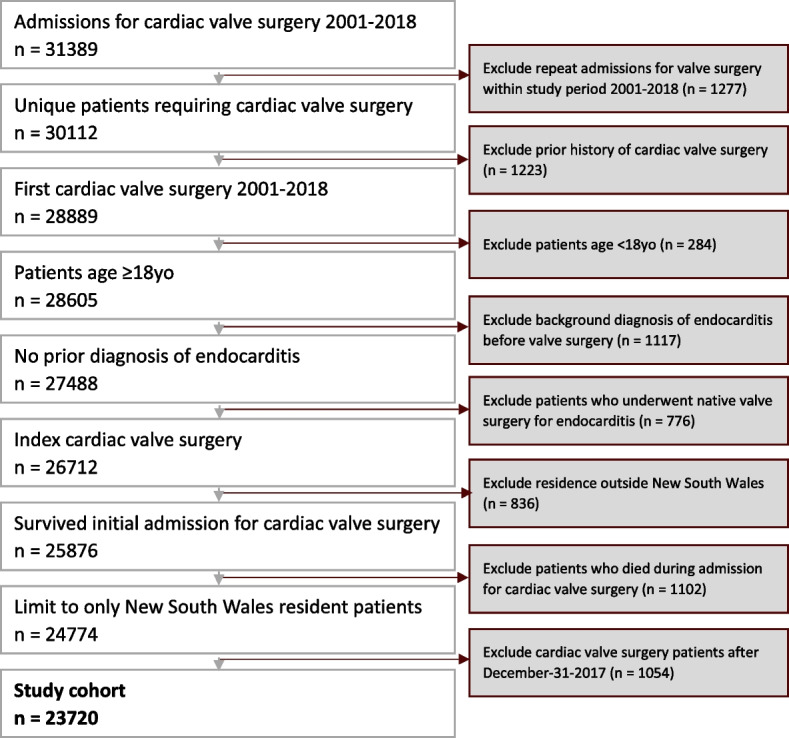
Derivation of study cohort

PVE was defined as any IE occurring in a new admission after a previous open-heart cardiac valve surgery. In patients with valve replacements, PVE would be essentially synonymous with prosthetic valve endocarditis since IE on native valves after valve operations is extremely rare and has not been reported in moderately sized case series [17, 18]. Patients < 18 years old or with a history of IE prior to cardiac valve surgery were excluded. Surgeries in the final year (i.e., 2018) of the APDC database and non-NSW residents were excluded to maximize follow-up. The end of study follow-up was December 31, 2018 (full inclusion and exclusion criteria shown in Fig. 1).

Comorbidities were coded according to the International Statistical Classification of Diseases and Related Health Problems, Tenth Revision, Australian Modification (ICD-10AM) codes (Additional file 1: Table S1). Procedures were coded according to the Australian Classification of Health Interventions (ACHI) and included the date of the procedure (Additional file 1: Table S2). The type of cardiac valve surgery was coded according to the valve involved (aortic, mitral, tricuspid, pulmonary, or multiple valves) and the material used (bioprosthetic, mechanical, or valve repair).

For this study, we examined several healthcare-related procedural exposures performed prior to admission for PVE. Variables selected in our regression analysis were based on clinical and biological plausibility for increased risk of PVE. In addition, we examined the risk of PVE after any healthcare exposures. Any healthcare exposures were defined as having an invasive procedure, hemodialysis, or hospitalization for 2 days or longer in the past 90 days, similarly to previous literature [19, 20]. Healthcare-associated PVE was defined as an admission for PVE within 90 days of any healthcare exposure as defined above [19, 20]. Invasive dental procedures were not investigated. The beginning and duration of admission were derived using a standardized method [12, 21].

Baseline characteristics were calculated and stratified according to the occurrence of PVE using standard nonparametric techniques. The risk of PVE after the first cardiac valve surgery was plotted over time, competing with death, according to the Fine-Gray test. The risk of death after PVE was quantified with cumulative incidence curves using the Kaplan-Meier approach.

Time-dependent multivariable Cox regression analysis with cause-specific hazards was performed to assess the risk for PVE. Healthcare-related procedures during hospital admissions after cardiac valve surgery were input as binary time-dependent covariates to calculate the risk of PVE for 90 days after the procedure of interest. Covariates were selected using bidirectional stepwise regression starting with an empty set and permitting forward selection if *p* < 0.05 and permitting backward elimination if *p* < 0.1 (Additional file 1: Table S3). Multicollinearity was assessed with the generalized variance inflation factor. Adherence to the proportional hazard assumption was assessed by chi-squared test of the Schoenfeld residuals as well as visual inspection of the Schoenfeld residuals against time. Where the proportional hazards assumption was not satisfied, analysis was repeated with variables stratified into appropriate time intervals both to ensure model assumptions were met and to inspect how risk factors varied over time [22]. Specifically, the coefficients of time-varying covariates were stratified according to time since index cardiac valve surgery, with stratification at the approximate inflection points of the Schoenfeld residuals plotted against time. This methodology is well established [23].

Time-independent multivariable Cox regression analysis was performed to calculate the risk of mortality after PVE diagnosis using the same methodology as described above, with the background comorbidities and other covariates in the multivariable model based on the index valve surgery admission.

**Fig. 2:**
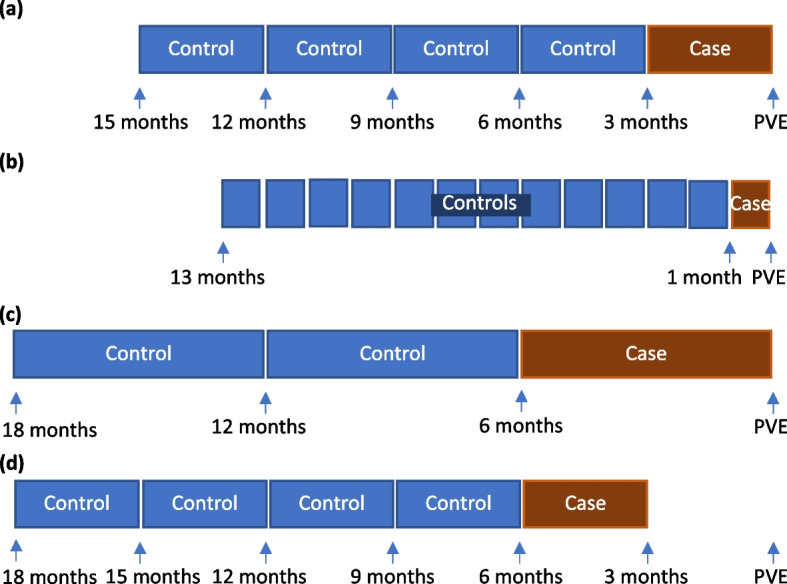
Case-crossover design with each patient acting as their own control. “At risk” period defined as **a** 1–90 days (3 months) after exposure (the standard definition), **b** 1–30 days (1 month) after exposure, **c** 1–180 days (6 months) after exposure, and **d** 90–180 days after exposure

To verify the robustness of the association between healthcare exposures and PVE for our study cohort, we repeated our analysis using a case-crossover design. Healthcare-related procedural exposures as described above and any healthcare exposures during the “at risk” time window of 1–90 days prior to PVE were compared to exposures during the previous year using conditional logistic regression. We then performed additional sensitivity analysis by varying the “at risk” time window from 1 to 90 days after exposure (standard definition) to (i) 1–30 days, (ii) 1–180 days, or (iii) 90–180 days after exposure (Fig. 2). This tested for a biologically plausible dose–response relationship between the recency of exposure and effect size, as has been tested in other epidemiological studies [14, 24]. The unadjusted incidence of hospitalization and procedures over time, prior to the diagnosis of PVE, was plotted. PVE diagnoses were also stratified according to microbiological diagnosis.

All analyses were performed using R version 4.3.0, with statistical significance set at *p* < 0.05 with the Bonferroni correction for multiple comparisons.

## Results

### Baseline characteristics and rates of PVE in the study cohort

**Fig. 3:**
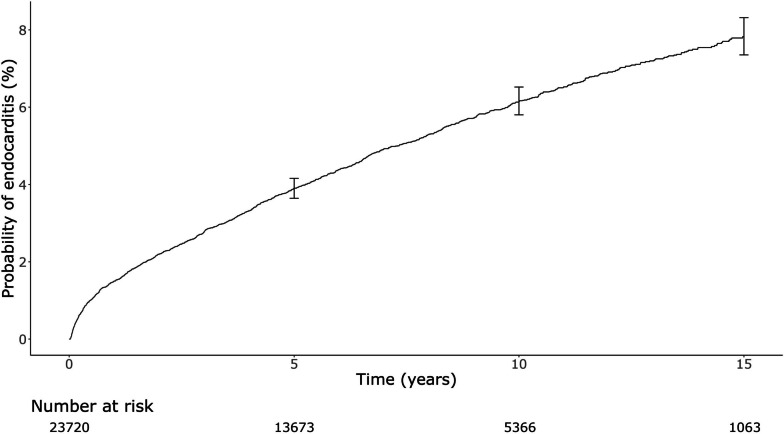
Cumulative incidence of PVE after cardiac valve surgery. Error bars indicate 95% confidence interval. The incidence of PVE was calculated by including death as a competing risk according to Fine-Gray’s test. PVE, post valve surgery endocarditis

The study cohort comprised 23,720 patients (63.4% male) who were discharged alive following cardiac valve surgery between 2001 and 2017 (Table 1). Of these, 1266 (5.3%) patients had a recorded episode of PVE during follow-up. The incidence of PVE was 1.5% after 1 year, 3.9% at 5 years, and 7.8% at 15 years (Fig. 3). The median age of the total cohort was 73 (IQR 65–79) years old and was slightly younger for patients who subsequently experienced PVE (Table 1). Male patients were significantly more likely to experience PVE. The median length of hospital stay during the index valve surgery was 13 (IQR 9–24) days and did not differ between those who did or did not subsequently develop PVE. Two thirds (65%) of patients with PVE had a transesophageal echocardiogram within 2 weeks of the admission date where PVE was diagnosed. Of patients with PVE, 16% (*n* = 197) had open-heart valve surgery within 90 days of diagnosis. For more detailed information on our study cohort’s baseline characteristics, please refer to Additional file 1: Table S4 and Table S5.

**Table 1:** Patient characteristics at time of cardiac valve surgery stratified by occurrence of subsequent PVE

**Clinical parameters at index cardiac valve surgery admission**	**Total cohort (*n* = 23,720)**	**PVE cohort (*n* = 1266)**	**No PVE cohort (*n* = 22,454)**	***p*-value**
Male	15,050 (63.4%)	873 (69%)	14,177 (63.1%)	< 0.001
Age, median years (IQR)	72.9 (64.7–79)	72 (62.9–78.2)	72.9 (64.9–79)	< 0.001
Private hospital	11,891 (50.1%)	601 (47.5%)	11,290 (50.3%)	0.055
Admitted from ED	18,361 (77.4%)	999 (78.9%)	17,362 (77.3%)	0.201
LOS, median days (IQR)	13 (9–24)	13 (9–23)	13 (9–24)	0.271
ICU admission	20,420 (86.1%)	1084 (85.6%)	19,336 (86.1%)	0.654

*ED* emergency department, *ICU* intensive care unit, *IQR* interquartile range, *LOS* length of stay, *PVE* post valve surgery endocarditis

### Association of PVE with healthcare-related exposures

Healthcare-associated PVE (i.e., healthcare exposure within 90 days) accounted for 37% of PVE (Table 2). The most common invasive procedures prior to PVE were red cell transfusions (accounting for 16% of pre-PVE healthcare exposures), gastroscopy (9%), colonoscopy (8%), coronary angiogram (7%), and hemodialysis (5%) (Table 2). Only 277 patients (22%) had no healthcare exposure at any time between index valve surgery and PVE (not shown).

**Table 2:** Frequency of healthcare exposures occurring prior to PVE (*n* = 1266)

**Exposure**	**Count within 30 days of PVE**^**a**^	**Count within 90 days of PVE**^**a**^	**Count within 6 months of PVE**^**a**^
Any healthcare exposure^b^	253 (20.0%)	473 (37.4%)	611 (48%)
Red cell transfusion	40 (3.2%)	75 (5.9%)	111 (8.8%)
Gastroscopy	18 (1.4%)	42 (3.3%)	67 (5.3%)
Colonoscopy	8 (0.6%)	39 (3.0%)	64 (5.1%)
Coronary angiogram	18 (1.4%)	35 (2.8%)	46 (3.6%)
Hemodialysis	23 (1.8%)	26 (2.1%)	30 (2.4%)
Pacing wire insertion	5 (0.4%)	16 (1.3%)	28 (2.2%)
Cystoscopy	6 (0.5%)	14 (1.1%)	29 (2.3%)
Central venous catheter insertion	4 (0.3%)	12 (0.9%	16 (1.3%)
Open-heart cardiac valve surgery^c^	3 (0.2%)	8 (0.6%)	9 (0.7%)
Skin biopsy	2 (0.2%)	4 (0.3%)	4 (0.3%)
Chemotherapy	1 (0.1%)	1 (0.1%)	2 (0.2%)
Bronchoscopy	0 (0%)	2 (0.2%)	2 (0.2%)
Coronary artery bypass graft	0 (0%)	0 (0%)	1 (0.1%)

^a^Each patient only counted once

^b^Represents any of the listed exposures (as surrogates for receiving intravenous therapy), or hospitalization for 2 days or longer

^c^Refers to repeat open-heart cardiac valve surgery after the index surgery

**Fig. 4:**
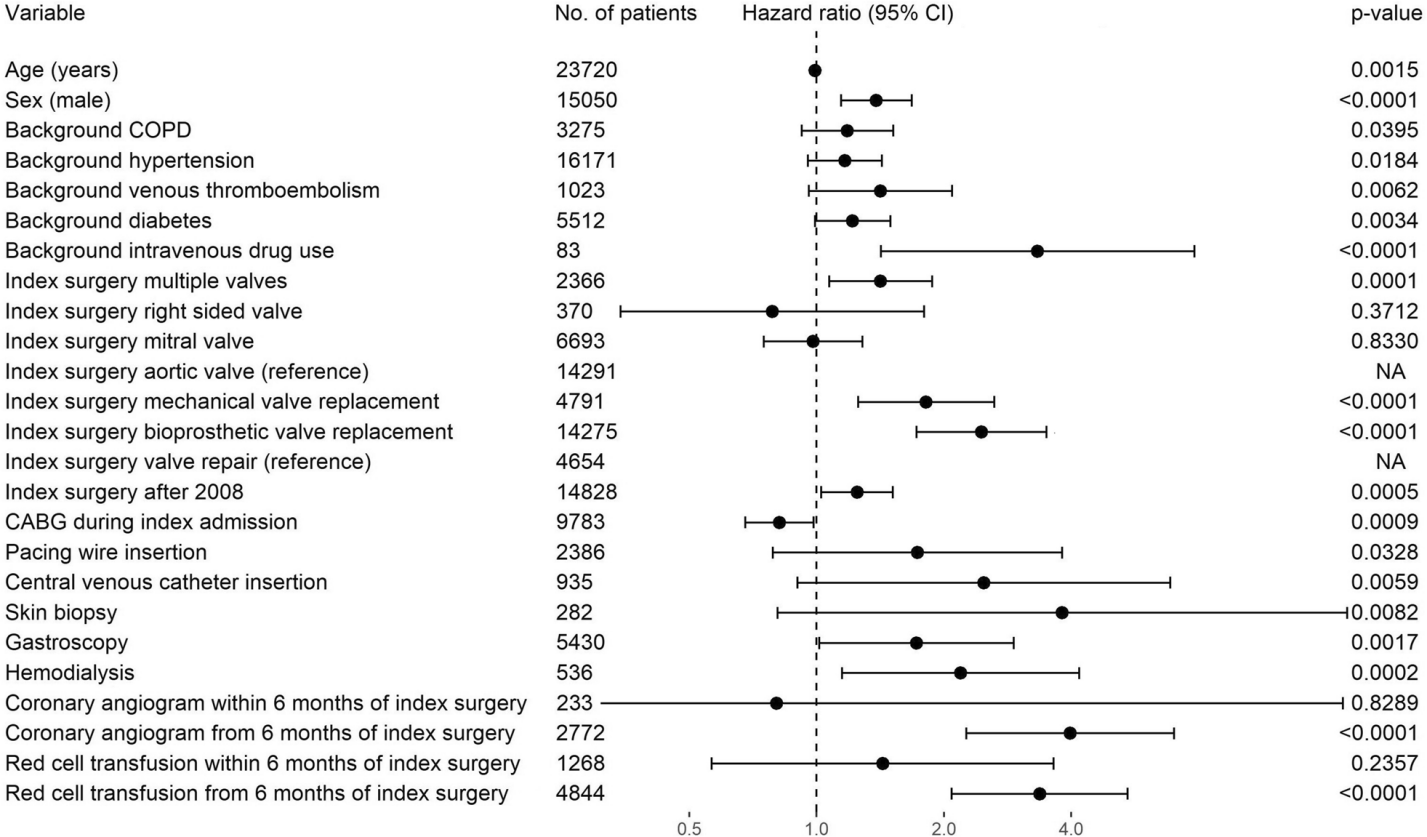
Hazard ratios for PVE based on multivariable Cox regression model. Hazard ratios for PVE after invasive procedures calculated for 90 day after the invasive procedure. *n* = 23,720. Bonferroni corrected critical *p*-value for multiple comparisons was 0.0022, and 95% confidence interval was Bonferroni adjusted. Index surgery after 2008 indicates after July 2008 (at the time of change in Australian antibiotic prescribing guidelines). CABG, coronary artery bypass surgery; COPD, chronic obstructive pulmonary disease; NA, not applicable; PVE, post cardiac valve surgery endocarditis

The risk of PVE was increased for 90 days after invasive procedures including hemodialysis, coronary angiography, and red cell transfusion (Fig. 4). Undergoing surgery involving multiple valves (hazard ratio [HR] 1.4, 95% confidence interval [CI] 1.1–1.9) and having a bioprosthetic valve replacement instead of valve repair (HR 2.5, 95% CI 1.7–3.5) were associated with a higher incidence of PVE, while having concomitant coronary artery bypass graft (CABG) surgery during index admission was associated with a lower risk of PVE (HR 0.8, 95% CI 0.7–0.98) (Fig. 4). Bioprosthetic valves were slightly more strongly associated with PVE than mechanical valves, although this difference was not significant after Bonferroni correction. A background of intravenous drug use (IVDU) (HR 3.3, 95% CI 1.4–7.8) and being male (HR 1.4, 95% CI 1.1–1.7) were associated with PVE. Having index valve surgery after 2008 was also associated with PVE (HR 1.2, 95% CI 1.03–1.5).

**Fig. 5:**
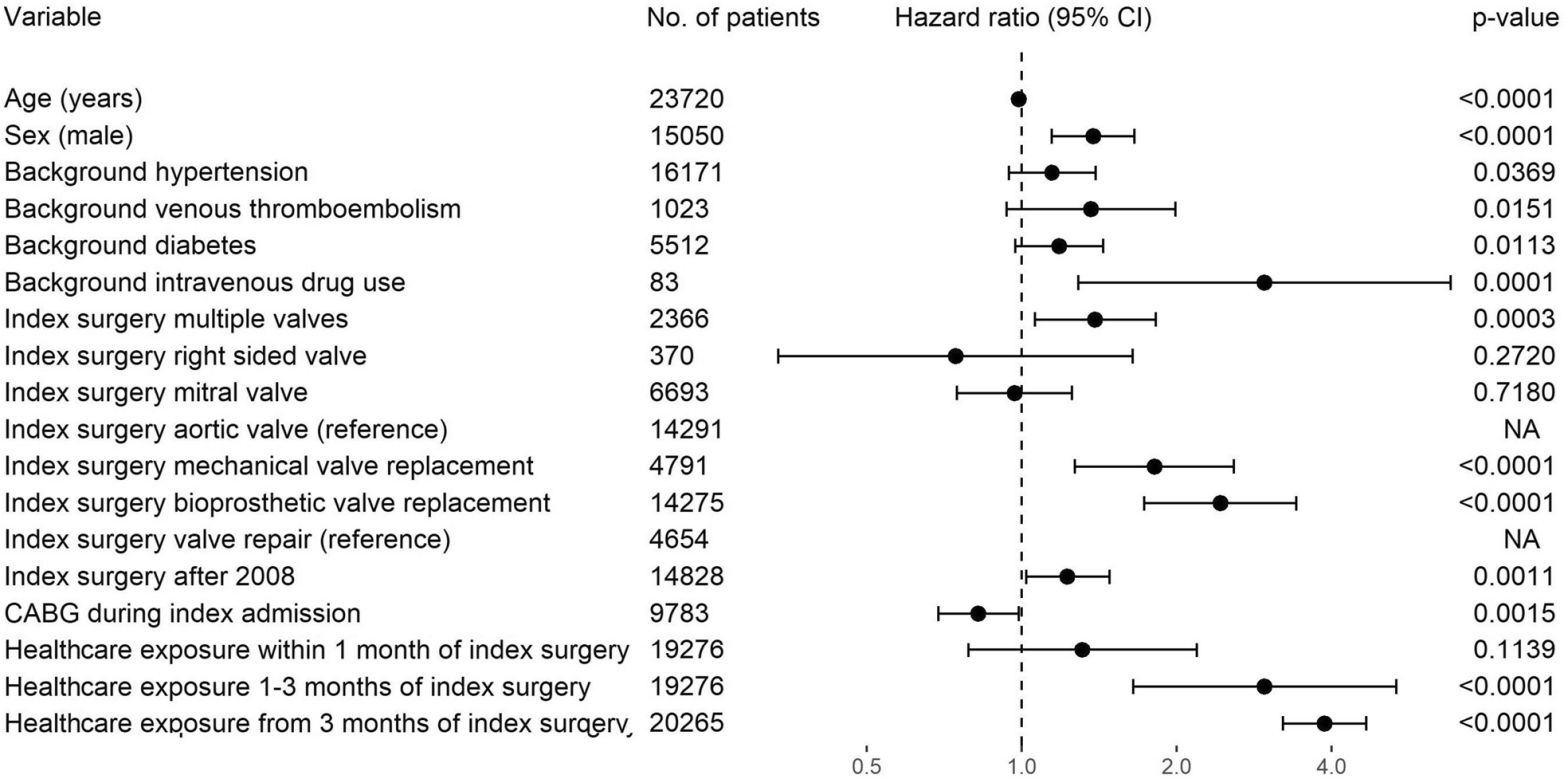
Hazard ratios for PVE based on multivariable Cox regression model including 90 day risk of PVE after any healthcare exposure. Figure shows the multivariable analysis based on any healthcare exposure defined as having an invasive procedure, hemodialysis, or hospitalization for 2 days or longer. *n* = 23,720. Bonferroni corrected critical *p*-value for multiple comparisons was 0.0031, and 95% confidence interval was Bonferroni adjusted. Index surgery after 2008 indicates after July 2008 (at the time of change in Australian antibiotic prescribing guidelines). CABG, coronary artery bypass surgery; CI, confidence interval; NA, not applicable; PVE, post cardiac valve surgery endocarditis. Excluding hemodialysis, a recurring exposure event from our definition of healthcare exposure made minimal change to the results: HR for PVE after healthcare exposure within 1 month of index valve surgery was 1.3 (95% CI 0.8–1.8) vs 1.3 (95% CI 0.95–1.9) if hemodialysis was excluded; HR after healthcare exposure 1–3 months after index valve surgery was 3.0 (95% CI 2.0–4.4) vs 2.8 (95% CI 1.9–4.2) if hemodialysis was excluded; HR after healthcare exposure > 3 months beyond index valve surgery was 3.9 (95% CI 3.4–4.4) vs 3.8 (95% CI 3.4–4.3) if hemodialysis was excluded

Healthcare exposures collectively were strongly associated with the risk of PVE. In general, the earlier the healthcare exposure occurred after index valve surgery, the weaker the association with PVE. For example, healthcare exposure within 30 days of index valve surgery was not associated with PVE (HR 1.3, 95% CI 0.8–2.2), whereas healthcare exposure greater than or equal to 90 days after index valve surgery was strongly associated with PVE (HR 3.9, 95% CI 3.2–4.7) (Fig. 5 and Additional file 1: Figure S1). Several procedures including red cell transfusion (HR 3.4, 95% CI 2.1–5.4) and coronary angiogram (HR 4.0, 95% CI 2.3–7.0) were risk factors for PVE, although if they occurred within 6 months of index cardiac valve surgery, they did not significantly associate with PVE (Fig. 4).

As indicated in Figs. 4 and 5, certain healthcare exposures were stratified according to the time they occurred relative to index cardiac valve surgery. The stratification of healthcare exposures according to time since index valve surgery was required to satisfy the proportional hazards assumption. When coronary angiogram, red cell transfusions, and healthcare exposures in general were input as time-dependent covariates into a multivariable Cox regression model (Additional file 1: Figure S2 and Figure S3), they violated the proportional hazards assumption (Additional file 1: Figures S4 and Figure S5), although not collinearity (Additional file 1: Table S6 and Table S7). After stratification of the above parameters according to the inflection points of the Schoenfeld residuals, all model assumptions were met including the proportional hazards assumption and absence of multicollinearity (Additional file 1: Figure S6 and Figure S7, Table S8 and Table S9).

The most common microbiological identification during PVE was of streptococcus (Additional file 1: Table S10). However, there was limited information on microbiological diagnosis in the present study with 63% of patients having no recorded microbial data.

### Sensitivity analysis of “at risk period” using case-crossover design

**Fig. 6:**
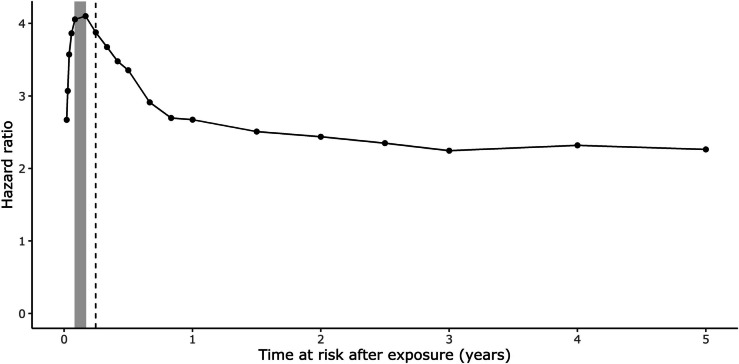
Hazard ratio for PVE after healthcare exposure according to duration considered to be at risk. Adjusted hazard ratios in multivariable Cox regression analysis using standard set of variables (as in Fig. 5). Reported hazard ratios are for healthcare exposures that occur beyond 3 months from index cardiac valve surgery. Vertical dotted line marks 90 days (the duration used in the present study). Shaded area indicates days 30–60

A sensitivity analysis was undertaken using a case-crossover design with each patient acting as their own pre-PVE control and varying the “at risk period” from 30 days to between 90 and 180 days (Fig. 2). This confirmed that several procedures were much more common in the 90 days prior to PVE diagnosis than in the year prior to this time window (Table 3). Exposure to coronary angiogram (odds ratio [OR] 3.9, 95% CI 2.4–6.4) and red cell transfusion (OR 2.6, 95% CI 1.8–3.8) were the most significant risk factors for PVE in the subsequent 90 days. In general, for procedures that showed a significant association with PVE in the time window of 90 days such as coronary angiogram, red cell transfusion, gastroscopy, and skin biopsy (but not central venous catheter), the more temporally proximate the healthcare exposures were to PVE, the stronger the relationship (Table 3). By contrast, there was minimal increase in the odds ratio of PVE if the healthcare-related procedural exposures occurred between 90 and 180 days before PVE. Graphically, one-off exposures such as red cell transfusion and coronary angiography were found to peak prior to PVE diagnosis, whereas recurring exposures such as hemodialysis produced a more gradual peak (Additional file 1: Figure S8). In Cox regression models, the risk of PVE was highest during the month after any healthcare exposure and declined over subsequent months to a plateau after 8 months (Fig. 6).

**Table 3:** Risk of PVE during different healthcare exposure time window periods prior to PVE^a^

**Type of exposures**	**Case: 1-30 days before PVE**		**Case: 1-90 days before PVE**		**Case: 1-180 days before PVE**		**Case: 90-180 days before PVE**	
	**Odds ratio**	***p*-value**	**Odds ratio**	***p*-value**	**Odds ratio**	***p*-value**	**Odds ratio**	***p*-value**
Coronary angiogram	4.4 (1.9-10.3)	< 0.0001	3.9 (1.8-8.1)	< 0.0001	2.9 (1.4-6)	< 0.0001	1 (0.3-3.1)	1
Red cell transfusions	4.3 (2.3-8.1)	< 0.0001	2.6 (1.5-4.6)	< 0.0001	2.5 (1.4-4.5)	< 0.0001	1.2 (0.6-2.5)	0.3948
Central venous catheter	1.9 (0.3-12.6)	0.2965	3.9 (1.1-14.5)	0.002	3.6 (0.9-14.3)	0.0059	1.9 (0.3-12.3)	0.3196
Gastroscopy	1.9 (0.8-4.7)	0.0322	1.9 (1-3.6)	0.005	1.6 (0.9-2.8)	0.0262	1.2 (0.5–2.5)	0.5845
Skin biopsy	8 (0.5-116.6)	0.0227	6 (0.4-87.5)	0.0497	3 (0.2-43.7)	0.2288	0 (NA)	0.9905
Pacing wire insertion	1.2 (0.2-7.5)	0.7201	2.1 (0.7-6.2)	0.0568	2.9 (1-8.3)	0.0032	2.2 (0.6-7.7)	0.0637
Colonoscopy	0.8 (0.2-2.6)	0.5053	1.5 (0.8-2.9)	0.0581	1.3 (0.7-2.3)	0.1826	1.2 (0.6-2.4)	0.5708
Hemodialysis	4.3 (0.8-24.9)	0.0142	2.8 (0.5-17.5)	0.0948	6.9 (0.7-71.2)	0.0151	4.2 (0.5-37)	0.0497
Open-heart cardiac valve surgery^b^	1.8 (0.2-17.2)	0.4196	2 (0.4-10)	0.2057	0.8 (0.2-3.6)	0.6181	0 (NA)	0.9898
Percutaneous coronary intervention	2.7 (0.3-28.2)	0.2066	2.1 (0.3-13.3)	0.248	1.5 (0.2-9)	0.5248	0.5 (0-11.2)	0.5134
Cystoscopy	1 (0.2-4.8)	0.9669	0.8 (0.3-2.5)	0.5581	1.2 (0.5-3.1)	0.5813	1.5 (0.5-4.1)	0.2657
Chemotherapy	0 (NA)	0.9902	0 (NA)	0.9905	2 (0-127)	0.624	4 (0.1-254)	0.327
Coronary artery bypass graft	0 (NA)	0.9908	0 (NA)	0.9924	0 (NA)	0.9939	0 (NA)	0.9924
Bronchoscopy	0 (NA)	0.9915	1 (0–26.6)	1	0.5 (0–13.3)	0.5353	0 (NA)	0.9913
Any healthcare exposure	2.7 (2-3.6)	< 0.0001	2.7 (2.1-3.7)	< 0.0001	2.6 (1.9-3.6)	< 0.0001	1.5 (1.1-2.1)	< 0.0001

*NA* not applicable, *PVE* post cardiac valve surgery endocarditis

^a^Based on case crossover design. Cases were compared to a control period of the preceding one year (Fig. 2). Conditional logistic regression method was used. Bonferroni corrected critical *p*-value for multiple comparisons was 0.0036, and 95% confidence interval was Bonferroni adjusted

^b^Refers to repeat open-heart cardiac valve surgery after the index surgery

### Mortality outcomes

**Fig. 7:**
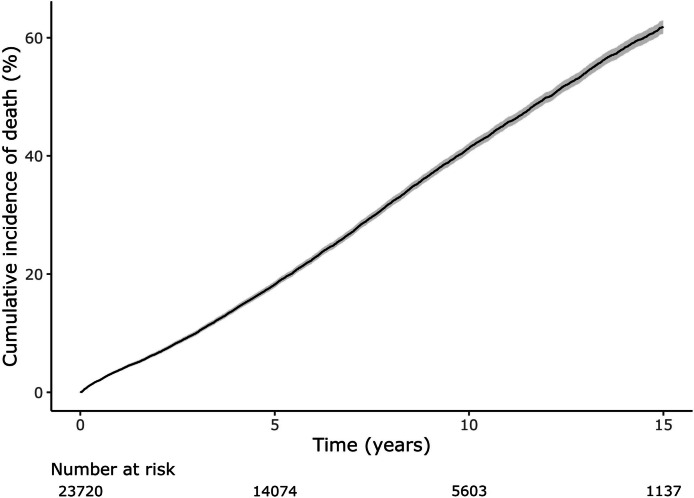
Cumulative incidence curve for mortality after open-heart valve surgery. Shaded area represents 95% confidence interval. Median follow-up 6.1 years. Kaplan-Meier approach

**Fig. 8:**
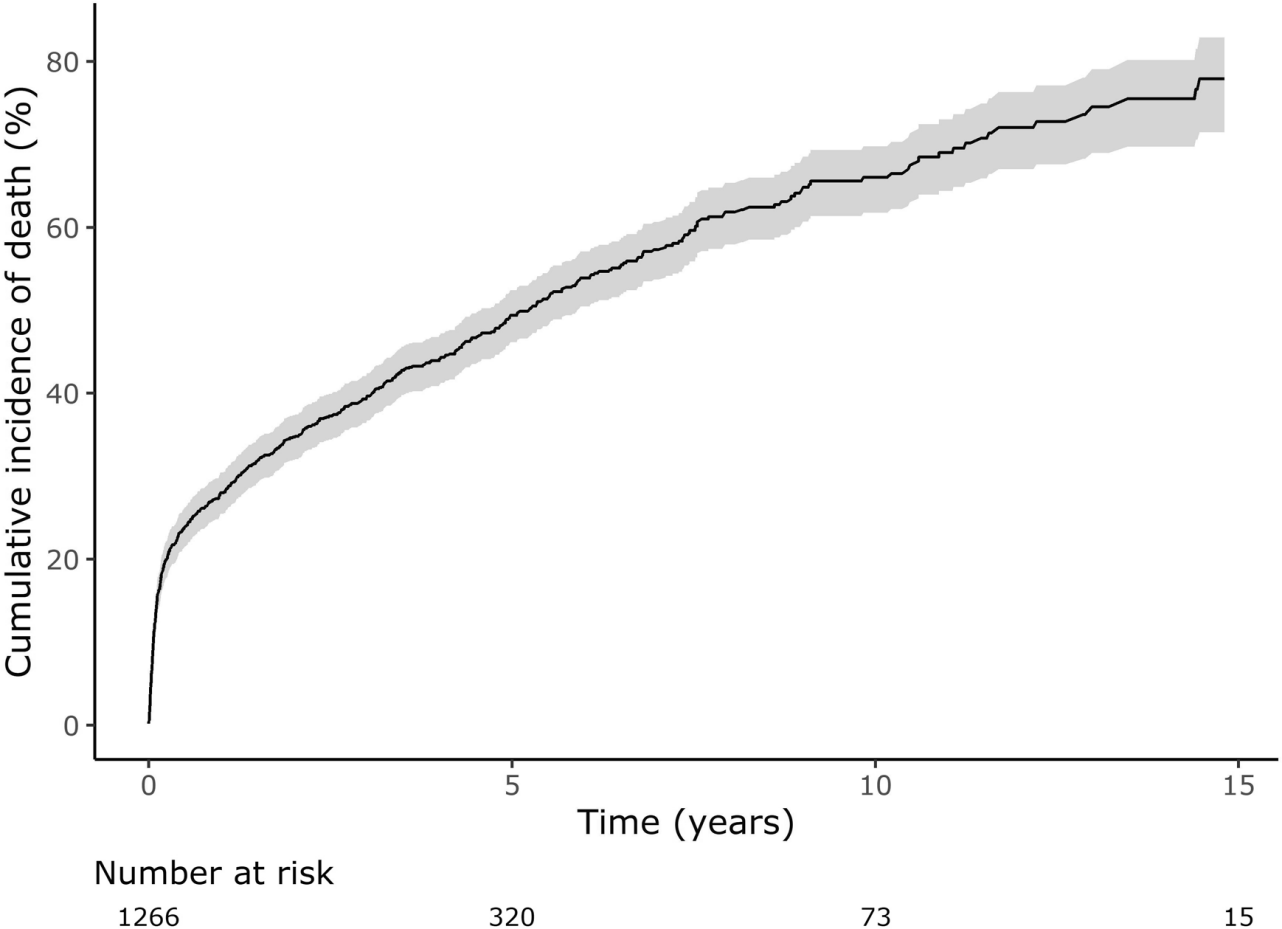
Cumulative incidence curve for mortality after PVE. Shaded area represents 95% confidence interval. Median follow-up 2.2 years. PVE, post cardiac valve surgery endocarditis. Kaplan-Meier approach

**Fig. 9:**
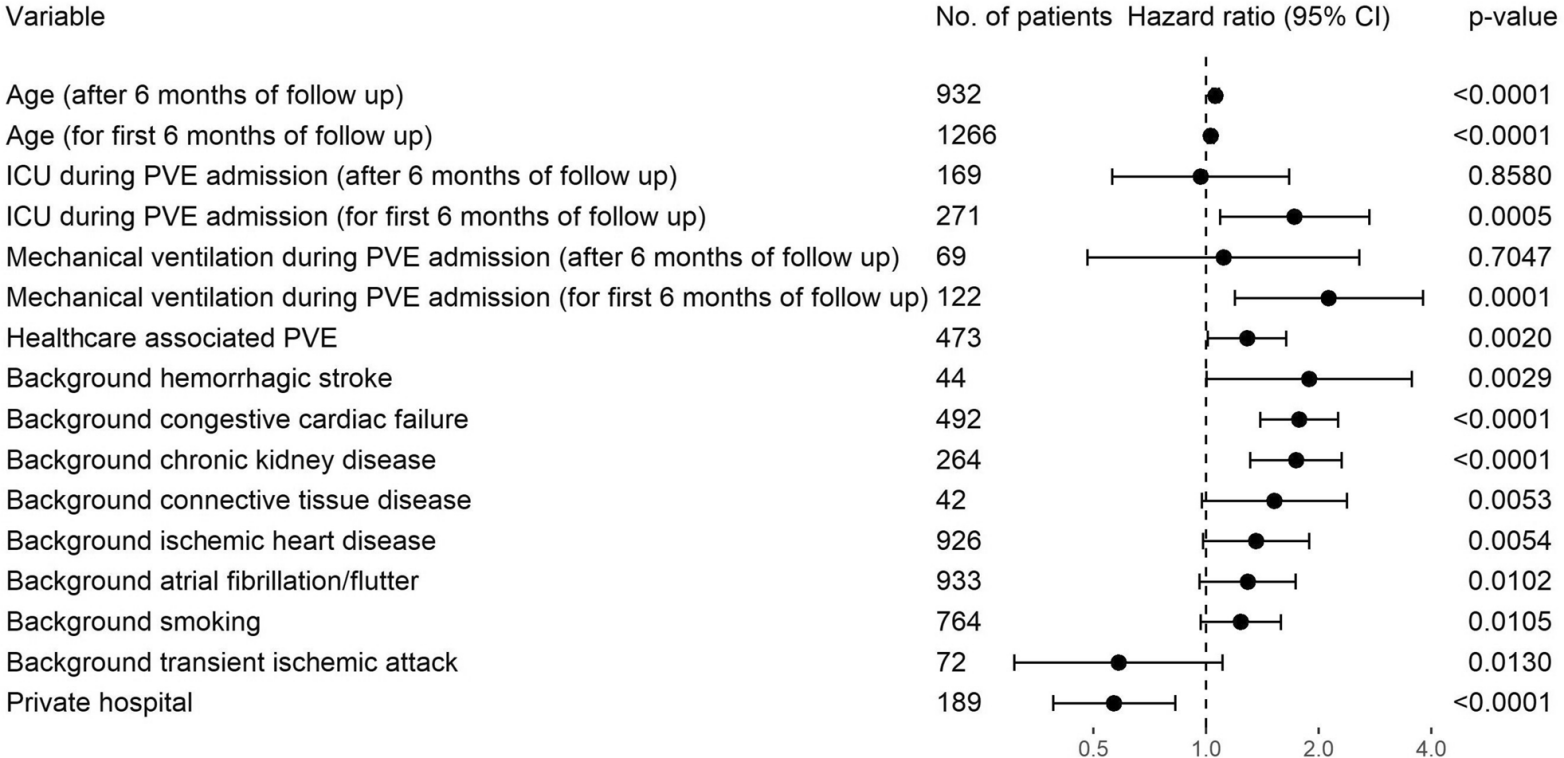
Hazard ratios for death from time of PVE calculated by multivariable Cox regression model. Bonferroni corrected critical *p*-value for multiple comparisons was 0.0031, and 95% confidence interval was Bonferroni adjusted. ICU, intensive care unit; PVE, post cardiac valve surgery

Patients who required cardiac valve surgery had a high mortality of approximately 4% every year (Fig. 7). By contrast, patients who experienced PVE had an even higher mortality especially in the immediate 3 months following diagnosis, during which 20% of patients died (Fig. 8). Patients with PVE were significantly more likely to die if their PVE was healthcare associated (HR 1.3, 95% CI 1.1–1.5) (Fig. 9).

## Discussion

We examined the risk factors for PVE and implications of healthcare exposures in a large state-wide cohort of patients who underwent open-heart cardiac valve surgery. The key findings from our study are as follows: (1) the 5-year cumulative risk for PVE after cardiac valve surgery was 3.9%; (2) PVE occurred more commonly after valve replacements, after surgeries involving multiple valves and in patients with specific baseline comorbidities including a history of IVDU and diabetes; (3) there was an approximately fourfold higher risk of PVE within 90 days of healthcare exposures such as red cell transfusions and coronary angiograms; and (4) healthcare-associated PVE accounted for more than one third of all PVE and was associated with a higher mortality than non-healthcare-associated PVE.

### Relationship with existing literature

Previous studies, albeit older series (all pre-2000), have identified risk factors for IE after cardiac valve surgery [25–29]. Similarly to the present study, these include being male and older age [25]. Most comorbidities that correlated with PVE in this study are well established for IE in general (although not PVE), including IVDU, diabetes, and hemodialysis [30–32]. In the present study, aortic and mitral valve replacements were associated with similar rates of PVE which is consistent with previous literature [25]. The association between multivalvular disease and PVE is also consistent with other literature [25, 26], although some conflicting evidence exists [27]. Patients with bioprosthetic valves were found to have a higher risk of PVE than those with mechanical valves; however, as with previous literature [28, 29], this was a non-significant finding.

Hospitalization and invasive procedures are recognized as risk factors for IE in general [33]. Several small case-control studies (fewer than 100 cases) have shown that specific perioperative healthcare exposures are associated with PVE such as any perioperative infection [34], transfusion of blood products [34], central line insertion [34], hemodialysis [34], repeat open-heart surgery [34], prolonged mechanical ventilation [26], wound infection [26], and gastrointestinal bleeding [35]. It is also well documented in studies only looking at dental exposures that invasive dental procedures are associated with a higher risk of IE in high-risk individuals [14], including those with prosthetic cardiac valves [36]. Two previous longitudinal studies have demonstrated that non-dental invasive procedures are temporally associated with IE including coronary angiography, bronchoscopy, and transfusion [13, 15]. Our large cohort study presents findings congruent with these previous studies and is also the first to utilize multivariable adjustment for confounders. It also presents data focused solely on PVE instead of IE in general.

Previous studies have indicated similar rates of healthcare-associated IE of approximately 30% [37–39], which is similar to the rate of healthcare-associated PVE in the present study of 37%. It has similarly been recognized that healthcare-associated IE is associated with higher mortality [39].

### Implications of study findings

The present study justifies additional caution for PVE in an already high-risk population by recognizing certain risk factors. The incidence of PVE is rising and indeed cardiac valve surgery after 2008 is an independent risk factor for the development of PVE. Our study showed that healthcare exposures, particularly involving red cell transfusion and coronary angiogram, are associated with a many-fold higher risk of PVE for the following 90 days and higher mortality after PVE.

Invasive investigation of the coronary arteries should be undertaken with caution, given an approximately four-fold higher risk of PVE. It has already been identified that many stable patients can have computed tomographic angiograms instead of invasive angiograms without the cost of additional cardiovascular events [40]. In the present study, revascularization during open-heart surgery via CABG was associated with a lower risk of PVE, which suggests that revascularization should be considered prior to or during cardiac valve surgery where indicated. Similarly, red cell transfusions should also be undertaken with caution. Recent literature tends to advocate for a more restrictive transfusion strategy, even in patients with significant cardiac disease [41].

This study is hypothesis-generating and supports future trials assessing the role of antibiotics prior to invasive procedures, particularly among those with other risk factors for PVE. For example, a patient with an annual 1% risk of PVE receiving a coronary angiogram that is associated with a fourfold higher risk of PVE for the next 90 days may be a good candidate to trial antibiotics (absolute risk of PVE within 90 days 1%, absolute risk of mortality within 6 months 0.3%). The present study is underpowered to recommend a specific time after index valve surgery that an invasive procedure should be performed if required. The trend that invasive procedures have less association with PVE when performed within 6 months of index valve surgery will require further studies to refute or verify this phenomenon.

### Study strengths and limitations

The present study has several strengths. Firstly, findings were based on a large cohort of over 20,000 patients with a follow-up of up to 17 years. Secondly, the major findings pertaining to the risk of PVE following healthcare exposures and invasive procedures were robust to model assumptions and replicated using completely different methodologies and multivariable adjustment.

Despite the above strengths, important limitations of our study should be highlighted. Firstly, data were not available regarding dental procedures, the use of antibiotics during healthcare exposures, or other high-risk groups who had not experienced prior cardiac valve surgery. Microbiological data were also largely lacking, which if available could have helped to infer its impact on mortality or link with health care associated PVE. Secondly, data regarding procedures performed in other Australian states were not available, although non-residents were omitted to maximize linkage analysis. Based on known emigration rates in NSW during our study period, the estimated non capture death is < 0.6% [42]. Thirdly, the diagnosis of IE was based on coded diagnoses, and clinical data were not available to validate this, although it was found that two-thirds of diagnoses of PVE had transesophageal echocardiogram at the time of diagnosis. Nevertheless, a previous study conducted in California, USA, showed that hospital coding of IE correlated well with endocarditis defined by Duke’s criteria (sensitivity 94%, specificity 99%) [19].

Finally, as this is an observational study, the direction of causality cannot be proven. As PVE is often a difficult and protracted diagnosis, procedures such as coronary angiography or red cell transfusions may be performed due to PVE symptoms but prior to the diagnosis of PVE being made. To limit reverse causality, we required that procedures or diagnoses considered as risk factors for PVE occurred during an episode of care prior to PVE diagnosis. Not only did possible risk factors have to occur before PVE, but the association was strongest between 30 and 60 days (Fig. 6) which is consistent with a biologically plausible incubation period.

## Conclusions

The epidemiology of PVE and risks of healthcare exposures in a high-risk population were investigated in this retrospective observational study that followed 23,720 patients for up to 17 years following open-heart cardiac valve surgery. In these patients, PVE was already common; however, healthcare exposures such as coronary angiography and red cell transfusion were associated with a much higher incidence and worse prognosis after PVE.

### Supplementary Information

**Additional file 1: Table S1.** Diagnostic categories by International Statistical Classification of Diseases and Related Health Problems, Tenth Revision, Australian Modifications (ICD-10AM). **Table S2.** Procedure categories by Australian Classification of Health Interventions (ACHI) code. **Table S3.** Risk of PVE in univariable Cox regression model for each covariate. **Table S4.** Characteristics of cardiac valve surgery stratified by occurrence of subsequent PVE. **Table S5.** Patient background medical history at time of cardiac valve surgery, stratified by occurrence of subsequent PVE. **Table S6.** Generalized variance inflation factors from multivariable regression model for risk of PVE (refer to Figure S2). **Table S7.** Generalized variance inflation factors from multivariable regression model for risk of PVE (refer to Figure S3). **Table S8.** Generalized variance inflation factors from multivariable regression model for risk of PVE (refer to Figure 4).**Table S9.** Generalized variance inflation factors from multivariable regression model for risk of PVE (refer to Figure 5). **Table S10.** Microbiological diagnosis at time of PVE.** Figure S1.** 90 -days risk of PVE following healthcare exposure, stratified by maximum time between healthcare exposure and index valve surgery^a^. **Figure S2.** Hazard ratios for PVE based on multivariable Cox regression model including 90 -day risk of PVE after invasive procedures. **Figure S3.** Hazard ratios for PVE based on multivariable Cox regression model including 90 -day risk of PVE after healthcare exposures. **Figure S4.** Schoenfeld residuals from multivariable regression model for risk of PVE (refer to Figure S2). **Figure S5.** Schoenfeld residuals from multivariable regression model for risk of PVE (refer to Figure S3). **Figure S6.** Schoenfeld residuals from multivariable regression model for risk of PVE (refer to Figure 4). **Figure S7.** Schoenfeld residuals from multivariable regression model for risk of PVE (refer to Figure 5). **Figure S8.** Incidence of healthcare exposures during 1 year prior to PVE in density plots.

## Data Availability

The datasets analyzed during the current study are available in the NSW Admitted Patient Data Collection and NSW Registry of Births Deaths & Marriages.

## Abbreviations

ACHI: Australian Classification of Health Interventions
APDC: Admitted Patient Data Collection
CABG: Coronary artery bypass graft
CHeReL: Centre for Health Record Linkage
ICD-10AM: International Statistical Classification of Diseases and Related Health Problems, Tenth Revision, Australian Modification
IE: Infective endocarditis
IVDU: Intravenous drug use
NSW: New South Wales
PVE: Post open-heart cardiac valve surgery endocarditis
