# Supplementary material for: Healthcare exposures and associated risk of endocarditis after open-heart cardiac valve surgery
Source: BMC Med. 2024 Feb 8;22:61. doi: 10.1186/s12916-024-03279-1 (PMC10854101; doi:10.1186/s12916-024-03279-1)
Supplement: Supplementary file 1 — Additional file 1: Table S1. Diagnostic categories by International Statistical Classification of Diseases and Related Health Problems, Tenth Revision, Australian Modifications (ICD-10AM). Table S2. Procedure categories by Australian Classification of Health Interventions (ACHI) code. Table S3. Risk of PVE in univariable Cox regression model for each covariate. Table S4. Characteristics of cardiac valve surgery stratified by occurrence of subsequent PVE. Table S5. Patient background medical history at time of cardiac valve surgery, stratified by occurrence of subsequent PVE. Table S6. Generalized variance inflation factors from multivariable regression model for risk of PVE (refer to Figure S2). Table S7. Generalized variance inflation factors from multivariable regression model for risk of PVE (refer to Figure S3). Table S8. Generalized variance inflation factors from multivariable regression model for risk of PVE (refer to Figure 4).Table S9. Generalized variance inflation factors from multivariable regression model for risk of PVE (refer to Figure 5). Table S10. Microbiological diagnosis at time of PVE. Figure S1. 90 -days risk of PVE following healthcare exposure, stratified by maximum time between healthcare exposure and index valve surgerya. Figure S2. Hazard ratios for PVE based on multivariable Cox regression model including 90 -day risk of PVE after invasive procedures. Figure S3. Hazard ratios for PVE based on multivariable Cox regression model including 90 -day risk of PVE after healthcare exposures. Figure S4. Schoenfeld residuals from multivariable regression model for risk of PVE (refer to Figure S2). Figure S5. Schoenfeld residuals from multivariable regression model for risk of PVE (refer to Figure S3). Figure S6. Schoenfeld residuals from multivariable regression model for risk of PVE (refer to Figure 4). Figure S7. Schoenfeld residuals from multivariable regression model for risk of PVE (refer to Figure 5). Figure S8. Incidence of healthcare exposu [file 12916_2024_3279_MOESM1_ESM.docx]

**Supplementary Information**

Kwan et al., Healthcare exposures and associated risk of endocarditis after open-heart cardiac valve surgery

**Table of Contents:**

**Table S1: Diagnostic categories by International Statistical Classification of Diseases and Related Health Problems, Tenth Revision, Australian Modifications (ICD-10AM)**

**Table S2: Procedure categories by Australian Classification of Health Interventions (ACHI) code**

**Table S3: Risk of PVE in univariable Cox regression model for each covariate**

**Table S4: Characteristics of cardiac valve surgery stratified by occurrence of subsequent PVE**

**Table S5: Patient background medical history at time of cardiac valve surgery, stratified by occurrence of subsequent PVE**

**Table S6: Generalized variance inflation factors from multivariable regression model for risk of PVE (refer to Figure S2)**

**Table S7: Generalized variance inflation factors from multivariable regression model for risk of PVE (refer to Figure S3)**

**Table S8: Generalized variance inflation factors from multivariable regression model for risk of PVE (refer to Figure 4)**

**Table S9: Generalized variance inflation factors from multivariable regression model for risk of PVE (refer to Figure 5)**

**Table S10: Microbiological diagnosis at time of PVE**

**Figure S1: 90-days risk of PVE following healthcare exposure, stratified by maximum time between healthcare exposure and index valve surgery^a^**

**Figure S2: Hazard ratios for PVE based on multivariable Cox regression model including 90-day risk of PVE after invasive procedures**

**Figure S3: Hazard ratios for PVE based on multivariable Cox regression model including 90-day risk of PVE after healthcare exposures**

**Figure S4: Schoenfeld residuals from multivariable regression model for risk of PVE (refer to Figure S2)**

**Figure S5: Schoenfeld residuals from multivariable regression model for risk of PVE (refer to Figure S3)**

**Figure S6: Schoenfeld residuals from multivariable regression model for risk of PVE (refer to Figure 4)**

**Figure S7: Schoenfeld residuals from multivariable regression model for risk of PVE (refer to Figure 5)**

**Figure S8: Incidence of healthcare exposures during 1 year prior to PVE in density plots**

**Table S1: Diagnostic categories by International Statistical Classification of Diseases and Related Health Problems, Tenth Revision, Australian Modifications (ICD-10AM)**

| **Morbidity** | **ICD-10AM codes** |
| --- | --- |
| Endocarditis | B37.6, I33, I33.0, I33.9, I38, I39, I39.0, I39.1, I39.2, I39.3, I39.4, I39.8 |
| History of prosthetic heart valve | Z95.2, Z95.3, Z95.4 |
| Rheumatic heart disease | I01, I01.0, I01.1, I01.2, I01.8, I01.9, I02.0, I05.1, I06, I06.0, I06.1, I06.2, I06.8, I06.9, I07, I09, I09.0, I09.1, I09.2, I09.8, I09.9 |
| Heart failure | I50.0, I11.0, I13.0, I13.2 |
| Atrial flutter or fibrillation | I48, I48.0, I48.1, I48.2, I48.3, I48.4, I48.9 |
| Ischemic stroke | G46, G46.0, G46.1, G46.2, G46.3, G46.4, G46.5, G46.6, G46.7, G46.8, I63, I63.0, I63.1, I63.2, I63.3, I63.4, I63.5, I63.6, I63.8, I63.9, I69.3 |
| Transient ischemic attack | G45, G45.0, G45.1, G45.2, G45.3, G45.4, G45.8, G45.9 |
| Hemorrhagic stroke | I60, I60.0, I60.1, I60.2, I60.3, I60.4, I60.5, I60.6, I60.7, I60.8, I60.9, I61, I61.0, I61.1, I61.2, I61.3, I61.4, I61.5, I61.6, I61.8, I61.9, I62, I62.0, I62.1, I62.9, I69.0, I69.1, I69.2 |
| Venous thromboembolism | I26, I26.0, I26.9, I80, I80.0, I80.1, I80.2, I80.3, I80.8, I80.9, I81, I82, I82.0, I82.1, I82.2, I82.3, I82.8, I82.9 |
| Pulmonary hypertension | I27.0, I27.2 |
| Ischemic heart disease | I20, I20.0, I20.1, I20.8, I20.9, I21, I21.0, I21.1, I21.2, I21.3, I21.4, I21.9, I22, I22.0, I22.1, I22.8, I22.9, I23, I23.0, I23.1, I23.2, I23.3, I23.4, I23.5, I23.6, I23.8, I24, I24.0, I24.1, I24.8, I24.9, I25, I25.0, I25.1, I25.10, I25.11, I25.12, I25.13, I25.2, I25.3, I25.4, I25.5, I25.6, I25.8, I25.9 |
| Previous coronary artery bypass graft | Z95.1, T82.2, T82.72, I25.12, I25.13 |
| Hypertension | I10, I11, I11.0, I11.9, I12, I12.0, I12.9, I13, I13.0, I13.1, I13.2, I13.9, I15, I15.0, I15.1, I15.2, I15.8, I15.9, U82.3 |
| Dyslipidemia | E78, E78.0, E78.1, E78.2, E78.3, E78.4, E78.5, E78.6, E78.8, E78.9 |
| Diabetes mellitus | E10, E10.0, E10.00, E10.01, E10.02, E10.1, E10.10, E10.11, E10.12, E10.13, E10.14, E10.15, E10.16, E10.2, E10.20, E10.21, E10.22, E10.23, E10.29, E10.3, E10.30, E10.31, E10.32, E10.33, E10.34, E10.35, E10.36, E10.39, E10.4, E10.40, E10.41, E10.42, E10.43, E10.49, E10.5, E10.50, E10.51, E10.52, E10.53, E10.59, E10.6, E10.60, E10.61, E10.62, E10.63, E10.64, E10.65, E10.69, E10.7, E10.70, E10.71, E10.73, E10.8, E10.80, E10.81, E10.9, E10.90, E10.91, E11, E11.0, E11.00, E11.01, E11.02, E11.1, E11.10, E11.11, E11.12, E11.13, E11.14, E11.15, E11.16, E11.2, E11.20, E11.21, E11.22, E11.23, E11.29, E11.3, E11.30, E11.31, E11.32, E11.33, E11.34, E11.35, E11.36, E11.39, E11.4, E11.40, E11.41, E11.42, E11.43, E11.49, E11.5, E11.50, E11.51, E11.52, E11.53, E11.59, E11.6, E11.60, E11.61, E11.62, E11.63, E11.64, E11.65, E11.69, E11.7, E11.70, E11.71, E11.72, E11.73, E11.8, E11.80, E11.81, E11.9, E11.90, E11.91, E12, E12.0, E12.00, E12.01, E12.1, E12.10, E12.11, E12.2, E12.20, E12.21, E12.3, E12.30, E12.31, E12.4, E12.40, E12.41, E12.5, E12.50, E12.51, E12.6, E12.60, E12.61, E12.7, E12.70, E12.71, E12.8, E12.80, E12.81, E12.9, E12.90, E12.91, E13, E13.0, E13.00, E13.01, E13.02, E13.1, E13.10, E13.11, E13.12, E13.13, E13.14, E13.15, E13.16, E13.2, E13.20, E13.21, E13.22, E13.23, E13.29, E13.3, E13.30, E13.31, E13.32, E13.33, E13.34, E13.35, E13.36, E13.39, E13.4, E13.40, E13.41, E13.42, E13.43, E13.49, E13.5, E13.50, E13.51, E13.52, E13.53, E13.59, E13.6, E13.60, E13.61, E13.62, E13.63, E13.64, E13.65, E13.69, E13.7, E13.70, E13.71, E13.72, E13.73, E13.8, E13.80, E13.81, E13.9, E13.90, E13.91, E14, E14.0, E14.00, E14.01, E14.02, E14.1, E14.10, E14.11, E14.12, E14.13, E14.14, E14.15, E14.16, E14.2, E14.20, E14.21, E14.22, E14.23, E14.29, E14.3, E14.30, E14.31, E14.32, E14.33, E14.34, E14.35, E14.36, E14.39, E14.4, E14.40, E14.41, E14.42, E14.43, E14.49, E14.5, E14.50, E14.51, E14.52, E14.53, E14.59, E14.6, E14.60, E14.61, E14.62, E14.63, E14.64, E14.65, E14.69, E14.7, E14.70, E14.71, E14.72, E14.73, E14.8, E14.80, E14.81, E14.9, E14.90, E14.91 |
| Current or ex-smoker | Z58.7, F17, F17.0, F17.2, F17.3, F17.4, F17.6, F17.7, F17.8, F17.9, T65.2, Z71.6, Z72.0, Z86.43 |
| Peripheral vascular disease | W09.01, E09.02, E09.5, E09.51, E09.52, E10.51, E10.52, E11.51, E11.52, E13.51, E13.52, E14.51, E14.52, I70, I70.0, I70.1, I70.2, I70.20, I70.21, I70.22, I70.23, I70.24, I70.8, I70.9, I71, I71.0, I71.00, I71.01, I71.02, I71.03, I71.1, I71.2, I71.3, I71.4, I71.5, I71.6, I71.8, I71.9, I72, I72.0 I72.1, I72.2, I72.3, I72.4, I72.5, I72.6, I72.8, I72.9, I73, I73.0, I73.1, I73.8, I73.9, I74, I74.0, I74.1, I74.2, I74.3, I74.4, I74.5, I74.8, I74.9, I77, I77.0, I77.1, I77.2, I77.3, I77.4, I77.5, I77.6, I77.8, I77.9, I78, I78.0, I78.1, I78.8, I78.9, I79, I79.0, I79.1, I79.2, I79.8 |
| Intravenous drug use | T40.0, T40.1, T40.2, T40.3, T40.4, T40.6, T43.61, F11, F11.0, F11.1, F11.2, F11.3, F11.4, F11.5, F11.6, F11.7, F11.8, F11.9, F15.0, F15.00, F15.01, F15.02, F15.09, F15.1, F15.10, F15.11, F15.12, F15.19, F15.2, F15.20, F15.21, F15.22, F15.29, F15.3, F15.30, F15.31, F15.32, F15.39, F15.4, F15.40, F15.41, F15.42, F15.49, F15.5, F15.50, F15.51, F15.52, F15.59, F15.6, F15.60, F15.61, F15.62, F15.69, F15.7, F15.70, F15.71, F15.72, F15.79, F15.8, F15.80, F15.81, F15.82, F15.89, F15.9, F15.90, F15.91, F15.92, F15.99 |
| Inflammatory bowel disease | K50, K50.0, K50.1, K50.8, K50.9, K51, K51.0, K51.2, K51.3, K51.4, K51.5, K51.8, K51.9, M07.4, M07.40, M07.41, M07.42, M07.43, M07.44, M07.45, M07.46, M07.47, M07.48, M07.49, M07.5, M07.50, M07.51, M07.52, M07.53, M07.54, M07.55, M07.56, M07.57, M07.58, M07.59, M07.6, M07.61, M07.62, M07.63, M07,64, M07.65, M07.66, M07.67, M07.68, M07.69, U84.1, U84.2, M09.1, M09.10, M09.11, M09.12, M09.13, M09.14, M09.15, M09.16, M09.17, M09.18, M09.19, M09.2, M09.20, M09.21, M09.22, M09.23, M09.24, M09.25, M09.26, M09.27, M09.28, M09.29 |
| Chronic obstructive pulmonary disease | J41, J41.0, J41.1, J41.8, J42, J43, J43.0, J43.1, J43.2, J43.8, J43.9, J44, J44.0, J44.1, J44.8, J44.9, J45, J45.0, J45.1, J45.8, J45.9, J46, J47, U83.1, U83.2, U83.3, J98.2, J98.3 |
| Connective tissue disorder | M30, M30.0, M30.1, M30.2, M30.3, M30.8, M31, M31.0, M31.1, M31.2, M31.3, M31.4, M31.5, M31.6, M31.7, M31.8, M31.9, M32, M32.0, M32.1, M32.8, M32.9, M33, M33.0, M33.1, M33.2. M33.9, M34, M34.0, M34.1, M34.2, M34.8, M34.9, M35, M35.0, M35.1, M35.2, M35.3, M35.4, M35.5, M35.6, M35.7, M35.8, M35.9, M36, M36.0, M36.1, M36.2, M36.3, M36.4, M36.8 |
| Chronic kidney disease | N18, N18.3, N18.4, N18.5, N18.9, N19, U87.1 |

**Table S2: Procedure categories by Australian Classification of Health Interventions (ACHI) code**

| **Procedures** | **ACHI codes** |
| --- | --- |
| All cardiac valve surgery | 38456-01, 38456-10, 38456-11, 38456-15, 38456-16, 38456-17, 38456-18, 38475-00, 38475-01, 38475-02, 38477-00, 38477-01, 38477-02, 38480-00, 38480-01, 38480-02, 38481-00, 38481-01, 38481-02, 38483-00, 38485-00, 38485-01, 38487-00, 38488-00, 38488-01, 38488-02, 38488-03, 38488-04, 38488-05, 38488-06, 38488-07, 38489-00, 38489-01, 38489-02, 38489-03, 38489-04, 38489-05, 38653-04, 38653-05, 38653-06, 38653-07, 38488-08 |
| Coronary artery bypass graft, including as combination with other procedure | 38456-19, 38497-00, 38497-01, 38497-02, 38497-03, 38497-04, 38497-05, 38497-06, 38497-07, 38500-00, 38500-01, 38500-02, 38500-03, 38500-04, 38500-05, 38503-00, 38503-01, 38503-02, 38503-03, 38503-04, 38503-05, 38637-00, 38653-08, 90201-00, 90201-01, 90201-02, 90201-03 |
| Percutaneous coronary intervention | 35304-00, 35305-00, 35310-00, 35310-01, 35310-02, 38300-00, 38303-00, 38306-00, 38306-01, 38306-02 |
| Coronary angiogram without angioplasty/stenting | 38200-00, 38203-00, 38206-00, 38215-00, 38218-00, 38218-01, 38218-02 |
| Permanent pacemaker or implantable cardioverter defibrillator or permanent temporary wire insertion | 38350-00, 38368-00, 38390-01, 38390-02, 38281-00, 38281-01, 38281-02, 38281-03, 38281-04, 38281-05, 38281-06, 38281-07, 38281-08, 38281-09, 38281-10, 38393-00, 38256-00, 38256-01 |
| Transoesophageal echocardiogram | 55118-00 |
| Bronchoscopy | 41898-00, 41898-01, 41898-02, 41904-00. 41892-01, 41901-00, 41898-03, 41892-01, 41901-00, 41889-00, 41892-00, 41904-00, 41892-01, 41901-00, 41895-00, 41892-01, 41901-00, 41889-01, 11500-00 |
| Colonoscopy | 32090-00, 32090-02, 32090-01, 90308-00, 90959-00, 90315-00, 32093-00, 90295-00, 90295-03, 32093-00, 90297-02, 32090-00, 32084-00, 32083-02, 32084-01, 90308-00, 90959-00, 90315-00, 32087-00, 32084-00 |
| Gastroscopy | 30473-00, 30478-07, 30473-07, 30476-03, 30473-01, 30478-01, 30478-04, 30478-02, 30478-07, 30478-03, 30476-03, 30478-00, 90296-00, 90297-01, 30473-02 |
| Cystoscopy | 36812-00, 36842-00, 90392-00, 36860-00, 36860-01, 36836-00, 37215-00, 36821-00, 36806-00, 36821-02, 36824-00, 36818-00, 36824-01, 36818-01, 36809-01, 37224-00, 36809-01, 37854-00, 36815-00, 36815-01, 36842-00, 90392-00, 36812-02, 37221-00, 36840-02, 36845-04, 36845-05, 36854-02, 37224-01, 36863-00, 36857-00, 36806-02, 36809-01, 36827-00, 36854-00, 36854-01, 36825-00, 36851-00, 37339-01, 36821-01, 36825-00, 36811-00, 36842-00, 36863-00, 36857-00, 36812-00, 36825-00, 36833-00, 36833-01, 36833-01, 36821-03, 36840-02, 36854-02, 36854-03, 36818-01, 36818-00, 36812-01, 36839-00, 36840-03, 36845-06, 36845-07, 37008-04 |
| Hemodialysis | 13100-00 |
| Red cell transfusion | 13706-02 |
| Chemotherapy | 35320-00, 35317-00, 35319-00, 35320-00, 13915-00, 13918-00, 13921-00 |
| Skin biopsy | 30071-0 |
| Aortic valve surgery | 38456-10, 38456-15, 38475-02, 38477-02, 38480-00, 38481-00, 38483-00, 38488-00, 38488-01, 38489-00, 38489-01, 38653-04 |
| Mitral valve surgery | 38456-16, 38475-00, 38477-00, 38480-01, 38481-01, 38485-00, 38485-01, 38487-00, 38488-02, 38488-03, 38489-02, 38653-05 |
| Tricuspid valve surgery | 38456-11, 38456-17, 38475-01, 38477-01, 38480-02, 38481-02, 38488-04, 38488-05, 38489-03, 38653-06 |
| Pulmonary valve surgery | 38456-01, 38456-18, 38488-06, 38488-07, 38489-04, 38489-05, 38653-07 |
| Mechanical valve insertion | 38488-00, 38488-02, 38488-04, 38488-06 |
| Bioprosthetic valve insertion | 38488-01, 38488-03, 38488-05, 38488-07, 38489-00, 38489-02, 38489-03, 38489-04, 38489-01, 38489-05, 38488-08 |

**Table S3: Risk of PVE in univariable Cox regression model for each covariate**

| **Variables^a^** | **Hazard ratio** | **p-value** |
| --- | --- | --- |
| Male | 1.32 (1.17-1.48) | <0.001 |
| Duration of index admission | 1 (1-1.01) | <0.001 |
| ICU admission | 1.23 (1.05-1.44) | 0.010 |
| Private hospital | 0.89 (0.8-0.99) | 0.035 |
| Age, years | 1 (0.99-1) | 0.525 |
| Admitted from ED | 0.98 (0.85-1.12) | 0.746 |
| Mechanical ventilation | 1.01 (0.9-1.13) | 0.838 |
| **Characteristics of index valve surgery** | | |
| Index surgery aortic valve (reference) | - | - |
| Index surgery mitral valve | 0.61 (0.54-0.71) | <0.001 |
| Index surgery right sided valve | 0.74 (0.45-1.23) | 0.247 |
| Index surgery 1ple valves | 1.2 (1.01-1.42) | 0.041 |
| Index surgery valve repair (reference) | - | - |
| Index surgery bioprosthetic valve replacement | 2.43 (2.02-2.92) | <0.001 |
| Index surgery mechanical valve replacement | 1.91 (1.55-2.35) | <0.001 |
| CABG during index surgery | 0.89 (0.79-1) | 0.043 |
| Index surgery after 2008^b^ | 1.33 (1.18-1.50) | <0.001 |
| **Background medical history** | | |
| Intravenous drug use | 4.06 (2.37-6.98) | <0.001 |
| Chronic kidney disease | 1.55 (1.27-1.9) | <0.001 |
| Venous thromboembolism | 1.55 (1.21-1.98) | <0.001 |
| Diabetes | 1.39 (1.22-1.57) | <0.001 |
| Hypertension | 1.27 (1.12-1.43) | <0.001 |
| Current or ex-smoker | 1.22 (1.1-1.37) | <0.001 |
| Chronic obstructive pulmonary disease | 1.29 (1.1-1.51) | 0.002 |
| Congestive cardiac failure | 1.22 (1.07-1.40) | 0.004 |
| Previous CABG | 1.24 (1.02-1.5) | 0.027 |
| Dyslipidemia | 1.1 (0.98-1.24) | 0.108 |
| Peripheral vascular disease | 1.12 (0.96-1.31) | 0.149 |
| Pulmonary hypertension | 1.12 (0.95-1.32) | 0.192 |
| Connective tissue disease | 1.29 (0.85-1.95) | 0.229 |
| Ischemic stroke | 1.19 (0.87-1.64) | 0.27 |
| Hemorrhagic stroke | 0.62 (0.23-1.64) | 0.332 |
| Transient ischemic attack | 0.88 (0.6-1.3) | 0.517 |
| Inflammatory bowel disease | 0.84 (0.4-1.77) | 0.646 |
| Ischemic heart disease | 0.98 (0.88-1.1) | 0.777 |
| Atrial fibrillation or flutter | 1.01 (0.9-1.13) | 0.819 |
| **Healthcare exposures** | | |
| Skin biopsy after index cardiac valve surgery | 6.89 (2.58-18.4) | <0.001 |
| Open-heart cardiac valve surgery after index cardiac valve surgery | 6.46 (3.21-12.97) | <0.001 |
| Central venous catheter insertion after index cardiac valve surgery | 5.43 (3.05-9.65) | <0.001 |
| Coronary angiogram after index cardiac valve surgery | 5.00 (3.57-7) | <0.001 |
| Red cell transfusion after index cardiac valve surgery | 4.16 (3.29-5.26) | <0.001 |
| **Variables^a^** | **Hazard ratio** | **p-value** |
| Hemodialysis after index cardiac valve surgery | 3.65 (2.46-5.42) | <0.001 |
| Gastroscopy after index cardiac valve surgery | 2.93 (2.17-3.97) | <0.001 |
| Pacing wire insertion after index cardiac valve surgery | 2.38 (1.44-3.93) | <0.001 |
| Colonoscopy after index cardiac valve surgery | 2.05 (1.5-2.82) | <0.001 |
| Coronary artery bypass graft after index cardiac valve surgery | 0 (NA) | <0.001 |
| Chemotherapy after index cardiac valve surgery | 6.28 (0.88-44.83) | 0.067 |
| Cystoscopy after index cardiac valve surgery | 1.54 (0.91-2.62) | 0.106 |
| Bronchoscopy after index cardiac valve surgery | 2.29 (0.57-9.21) | 0.242 |
| Any healthcare exposure | 3.54 (3.14-4.00) | <0.001 |

CABG = coronary artery bypass surgery; ED = Emergency Department; ICU = intensive care unit; IQR = interquartile range; LOS = length of stay; PVE = post cardiac valve surgery endocarditis

1. Each of the variables in this table were considered for inclusion in multivariable Cox regression models in the present study (Figure 4 and Figure 5)
2. Index surgery after 2008 indicates after July 2008 (at the time of change in Australian antibiotic prescribing guidelines)

**Table S4: Characteristics of cardiac valve surgery stratified by occurrence of subsequent PVE**

| **Cardiac valve surgery characteristics** | **Total cohort**  **(n=23720)** | **PVE cohort**  **(n=1266)** | **No PVE cohort**  **(n=22454)** | **p-value** |
| --- | --- | --- | --- | --- |
| Aortic valve surgery^a^ | 14291 (60.2%) | 833 (65.8%) | 13458 (59.9%) | <0.001 |
| Mitral valve surgery^a^ | 6693 (28.2%) | 265 (20.9%) | 6428 (28.6%) | <0.001 |
| Tricuspid valve surgery^a^ | 266 (1.1%) | 7 (0.6%) | 259 (1.2%) | 0.066 |
| Pulmonary valve surgery^a^ | 91 (0.4%) | 8 (0.6%) | 83 (0.4%) | 0.217 |
| Multiple valve surgery | 2379 (10%) | 153 (12.1%) | 2226 (9.9%) | 0.014 |
| Bioprosthetic valve insertion^b^ | 14275 (60.2%) | 843 (66.6%) | 13432 (59.8%) | <0.001 |
| Mechanical valve insertion^b^ | 4791 (20.2%) | 292 (23.1%) | 4499 (20%) | 0.010 |
| Valve repair | 4654 (19.6%) | 131 (10.3%) | 4523 (20.1%) | <0.001 |
| CABG during index surgery | 9783 (41.2%) | 478 (37.8%) | 9305 (41.4%) | 0.01 |

CABG = coronary artery bypass surgery; PVE = post valve surgery endocarditis

1. Single valve surgery (therefore mutually exclusive with multiple valve surgery)
2. Patients with bioprosthetic and mechanical valve inserted during index admission (n=38) were classified as mechanical valves

**Table S5: Patient background medical history at time of cardiac valve surgery, stratified by occurrence of subsequent PVE**

| **Background at cardiac valve surgery admission** | **Total cohort**  **(n=23720)** | **PVE cohort**  **(n=1266)** | **No PVE cohort**  **(n=22454)** | **p-value** |
| --- | --- | --- | --- | --- |
| Intravenous drug use | 83 (0.3%) | 14 (1.1%) | 69 (0.3%) | <0.001 |
| Dyslipidemia | 6628 (27.9%) | 411 (32.5%) | 6217 (27.7%) | <0.001 |
| Diabetes mellitus | 5512 (23.2%) | 334 (26.4%) | 5178 (23.1%) | 0.007 |
| Ischemic heart disease | 16297 (68.7%) | 833 (65.8%) | 15464 (68.9%) | 0.024 |
| Coronary stent present | 2272 (9.6%) | 103 (8.1%) | 2169 (9.7%) | 0.081 |
| Implantable cardiac device present | 1295 (5.5%) | 89 (7.0%) | 1206 (5.4%) | 0.014 |
| Atrial flutter or fibrillation | 15115 (63.7%) | 772 (61%) | 14343 (63.9%) | 0.040 |
| Current or ex-smoker | 12439 (52.4%) | 699 (55.2%) | 11740 (52.3%) | 0.045 |
| Rheumatic heart disease | 441 (1.9%) | 33 (2.6%) | 408 (1.8%) | 0.055 |
| Venous thromboembolism | 1023 (4.3%) | 67 (5.3%) | 956 (4.3%) | 0.091 |
| Transient ischemic attack | 641 (2.7%) | 26 (2.1%) | 615 (2.7%) | 0.169 |
| Hemorrhagic stroke | 151 (0.6%) | 4 (0.3%) | 147 (0.7%) | 0.196 |
| Previous CABG | 1928 (8.1%) | 115 (9.1%) | 1813 (8.1%) | 0.220 |
| Pulmonary hypertension | 2727 (11.5%) | 156 (12.3%) | 2571 (11.5%) | 0.367 |
| Hypertension | 16171 (68.2%) | 877 (69.3%) | 15294 (68.1%) | 0.406 |
| Inflammatory bowel disease | 188 (0.8%) | 7 (0.6%) | 181 (0.8%) | 0.409 |
| Heart failure | 4604 (19.4%) | 254 (20.1%) | 4350 (19.4%) | 0.570 |
| Chronic obstructive pulmonary disease | 3275 (13.8%) | 180 (14.2%) | 3095 (13.8%) | 0.694 |
| Connective tissue disorder | 394 (1.7%) | 23 (1.8%) | 371 (1.7%) | 0.740 |
| Peripheral vascular disease | 3484 (14.7%) | 188 (14.8%) | 3296 (14.7%) | 0.899 |
| Ischemic stroke | 772 (3.3%) | 40 (3.2%) | 732 (3.3%) | 0.909 |
| Chronic kidney disease | 1986 (8.4%) | 106 (8.4%) | 1880 (8.4%) | 1 |
| Human immunodeficiency virus | 39 (0.2%) | 3 (0.2%) | 36 (0.2) | 0.765 |

CABG = coronary artery bypass surgery; PVE = post valve surgery endocarditis

**Table S6: Generalized variance inflation factors from multivariable regression model for risk of PVE (refer to Figure S2)**

| **Variables** | **Degrees of freedom (df)** | $\sqrt[\mathbf{2df}]{\boldsymbol{GVIF}}$ |
| --- | --- | --- |
| Red cell transfusion | 1 | 1.23 |
| Coronary angiogram | 1 | 1.05 |
| Hemodialysis | 1 | 1.07 |
| Gastroscopy | 1 | 1.1 |
| Skin biopsy | 1 | 1.03 |
| Central venous catheter insertion | 1 | 1.13 |
| Pacing wire insertion | 1 | 1.02 |
| CABG during index admission | 1 | 1.05 |
| Index surgery after 2008 | 1 | 1.04 |
| Material in index surgery (bioprosthetic/mechanical/repair) | 2 | 1.16 |
| Valve in index surgery (AV/MV/right/multi) | 3 | 1.1 |
| Background intravenous drug use | 1 | 1.02 |
| Background diabetes | 1 | 1.06 |
| Background venous thromboembolism | 1 | 1.02 |
| Background hypertension | 1 | 1.08 |
| Background COPD | 1 | 1.02 |
| Sex (male) | 1 | 1.04 |
| Age (years) | 1 | 1.15 |

AV = aortic valve; CABG = coronary artery bypass graft; COPD = chronic obstructive pulmonary disease; MV = mitral valve; PVE = post cardiac valve surgery endocarditis

**Table S7: Generalized variance inflation factors from multivariable regression model for risk of PVE (refer to Figure S3)**

| **Variables** | **Degrees of freedom (df)** | $\sqrt[\mathbf{2df}]{\boldsymbol{GVIF}}$ |
| --- | --- | --- |
| Healthcare exposure | 1 | 1.03 |
| CABG during index admission | 1 | 1.05 |
| Index surgery after 2008 | 1 | 1.03 |
| Material in index surgery (bioprosthetic/mechanical/repair) | 2 | 1.16 |
| Valve in index surgery (AV/MV/right/multi) | 3 | 1.09 |
| Background intravenous drug use | 1 | 1.02 |
| Background diabetes | 1 | 1.05 |
| Background venous thromboembolism | 1 | 1.02 |
| Background hypertension | 1 | 1.08 |
| Sex (male) | 1 | 1.04 |
| Age (years) | 1 | 1.16 |

AV = aortic valve; CABG = coronary artery bypass graft; MV = mitral valve; PVE = post cardiac valve surgery endocarditis

**Table S8: Generalized variance inflation factors from multivariable regression model for risk of PVE (refer to Figure 4)**

| **Variables** | **Degrees of freedom (df)** | $\sqrt[\mathbf{2df}]{\boldsymbol{GVIF}}$ |
| --- | --- | --- |
| Red cell transfusion | 2 | 1.14 |
| Coronary angiogram | 2 | 1.04 |
| Hemodialysis | 1 | 1.07 |
| Gastroscopy | 1 | 1.1 |
| Skin biopsy | 1 | 1.02 |
| Central venous catheter insertion | 1 | 1.16 |
| Pacing wire insertion | 1 | 1.03 |
| CABG during index admission | 1 | 1.05 |
| Index surgery after 2008 | 1 | 1.04 |
| Material in index surgery (bioprosthetic/mechanical/repair) | 2 | 1.16 |
| Valve in index surgery (AV/MV/right/multi) | 3 | 1.1 |
| Background intravenous drug use | 1 | 1.02 |
| Background diabetes | 1 | 1.06 |
| Background venous thromboembolism | 1 | 1.03 |
| Background hypertension | 1 | 1.08 |
| Background COPD | 1 | 1.02 |
| Sex (male) | 1 | 1.04 |
| Age (years) | 1 | 1.15 |

AV = aortic valve; CABG = coronary artery bypass graft; COPD = chronic obstructive pulmonary disease; MV = mitral valve; PVE = post cardiac valve surgery endocarditis

**Table S9: Generalized variance inflation factors from multivariable regression model for risk of PVE (refer to Figure 5)**

| **Variables** | **Degrees of freedom (df)** | $\sqrt[\mathbf{2df}]{\boldsymbol{GVIF}}$ |
| --- | --- | --- |
| Healthcare exposure | 3 | 1.01 |
| CABG during index admission | 1 | 1.05 |
| Index surgery after 2008 | 1 | 1.03 |
| Material in index surgery (bioprosthetic/mechanical/repair) | 2 | 1.16 |
| Valve in index surgery (AV/MV/right/multi) | 3 | 1.09 |
| Background intravenous drug use | 1 | 1.02 |
| Background diabetes | 1 | 1.05 |
| Background venous thromboembolism | 1 | 1.02 |
| Background hypertension | 1 | 1.09 |
| Sex (male) | 1 | 1.04 |
| Age (years) | 1 | 1.16 |

AV = aortic valve; CABG = coronary artery bypass graft; MV = mitral valve; PVE = post cardiac valve surgery endocarditis

**Table S10: Microbiological diagnosis at time of PVE**

| **Microbiology** | **n (%)** |
| --- | --- |
| Not recorded | 793 (63) |
| Bacteraemia but without recorded identification | 96 (7.6) |
| *Streptococcus* | 173 (14) |
| *Staphylococcus aureus* | 31 (2.4) |
| Other *staphylococcus* species | 137 (11) |
| Gram negative bacteria | 22 (1.7) |
| *Candida* | 4 (0.32) |
| *Haemophilus* | 2 (0.16) |
| Polymicrobial | 8 (0.63) |

PVE = post cardiac valve surgery endocarditis

**Figure S1: 90-days risk of PVE following healthcare exposure, stratified by maximum time between healthcare exposure and index valve surgery^a^**


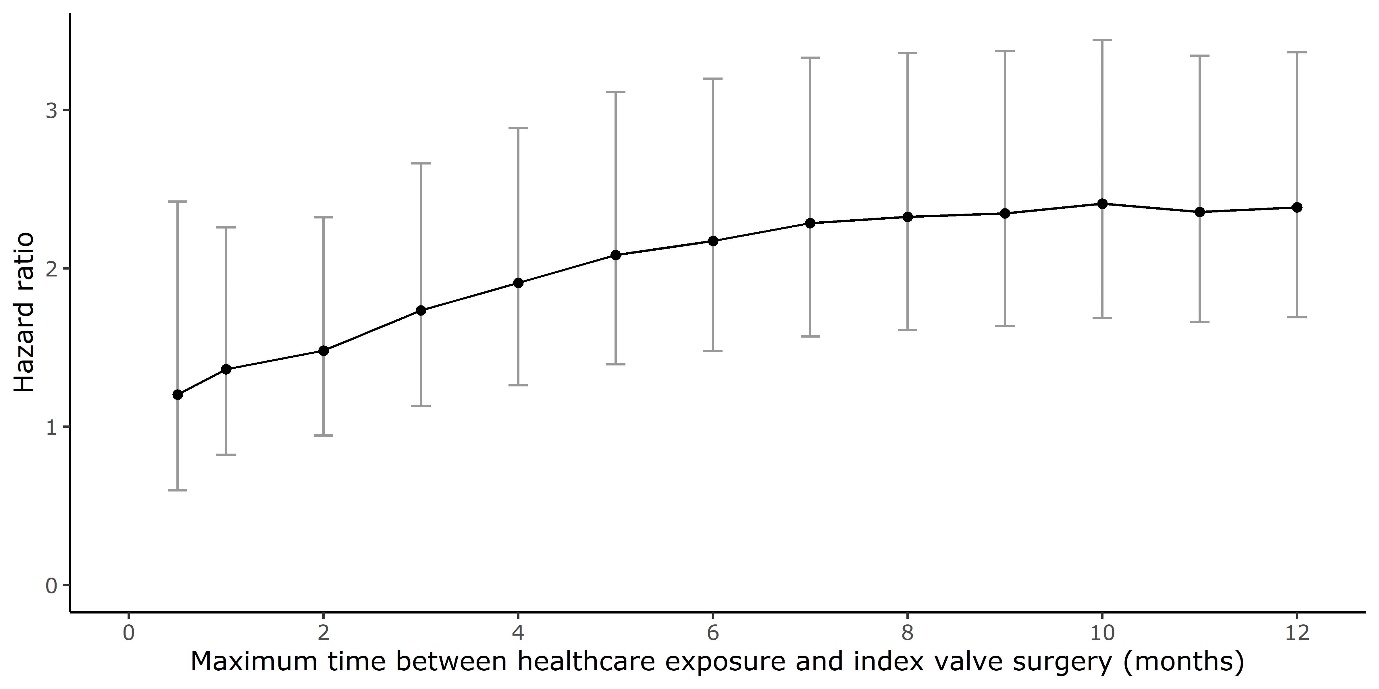
 **Figure Legend**

Figure shows the adjusted hazard ratio for the 90-days risk of PVE following healthcare exposure that occurred at specific time points following index valve surgery.

1. Each data point shown in the figure is derived from a different Cox regression model with multivariable adjustment for age, sex, comorbidities (hypertension, venous thromboembolism, diabetes and intravenous drug use), valve involved in index cardiac valve surgery, material used in index surgery, coronary artery bypass graft surgery during index surgery and index surgery occurring after July 2008 (as in Figure 5). Errorbars represent 95% confidence intervals with Bonferroni correction.

**Figure S2: Hazard ratios for PVE based on multivariable Cox regression model including 90-day risk of PVE after invasive procedures**

**
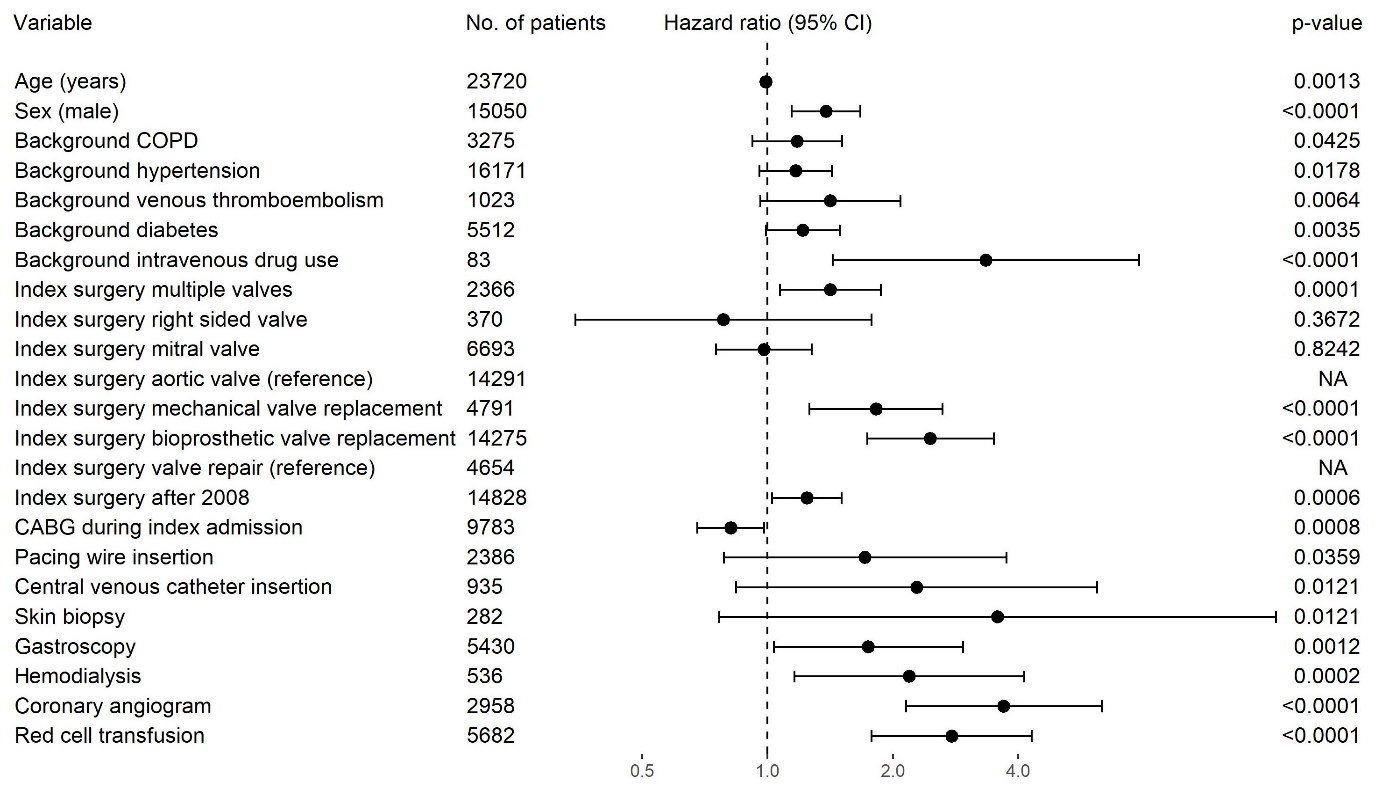
**

**Figure Legend**

Bonferroni corrected critical p-value for multiple comparisons was 0.0024, and 95% confidence interval was Bonferroni adjusted.
NB: proportional hazards assumption violated (see Figure S4)
Index surgery after 2008 indicates after July 2008 (at the time of change in Australian antibiotic prescribing guidelines)
CABG = coronary artery bypass surgery; COPD = chronic obstructive pulmonary disease; PVE = post cardiac valve surgery endocarditis

**Figure S3: Hazard ratios for PVE based on multivariable Cox regression model including 90-day risk of PVE after healthcare exposures**

**
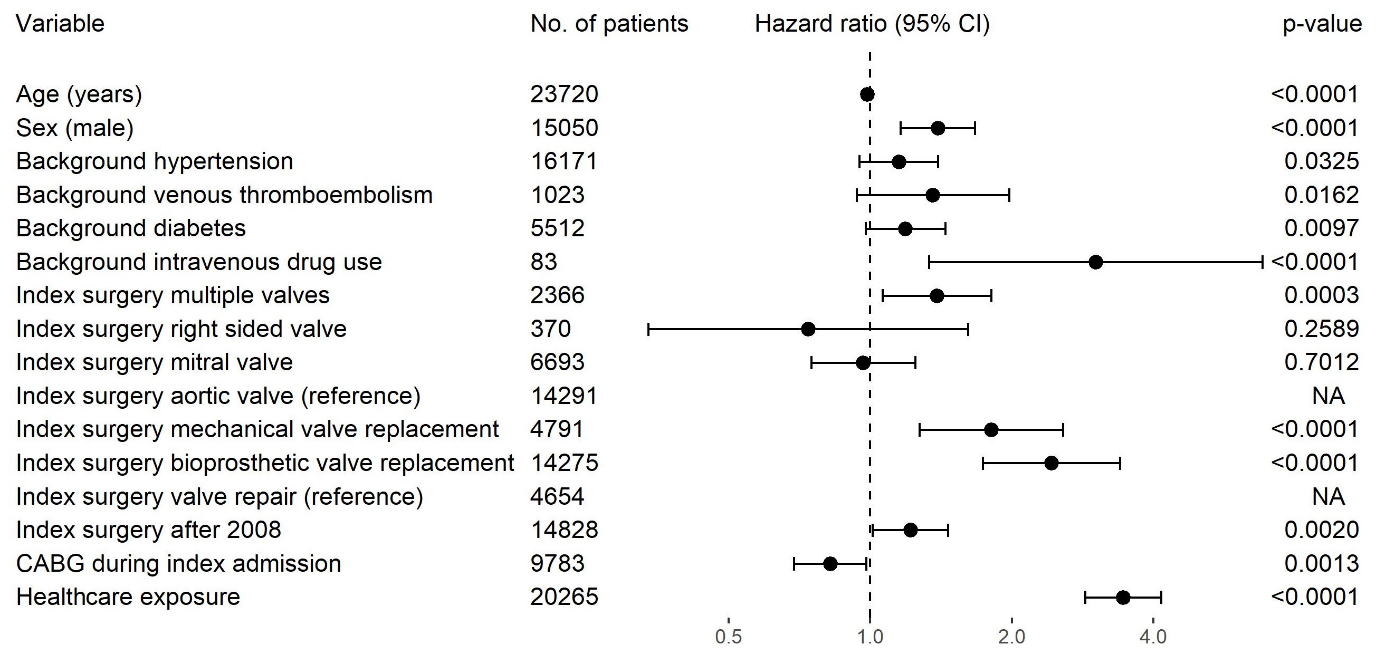
**

**Figure Legend**

Bonferroni corrected critical p-value for multiple comparisons was 0.0036, and 95% confidence interval was Bonferroni adjusted.
Index surgery after 2008 indicates after July 2008 (at the time of change in Australian antibiotic prescribing guidelines)
NB: proportional hazards assumption violated (see Figure S5)

**Figure S4: Schoenfeld residuals from multivariable regression model for risk of PVE (refer to Figure S2)**


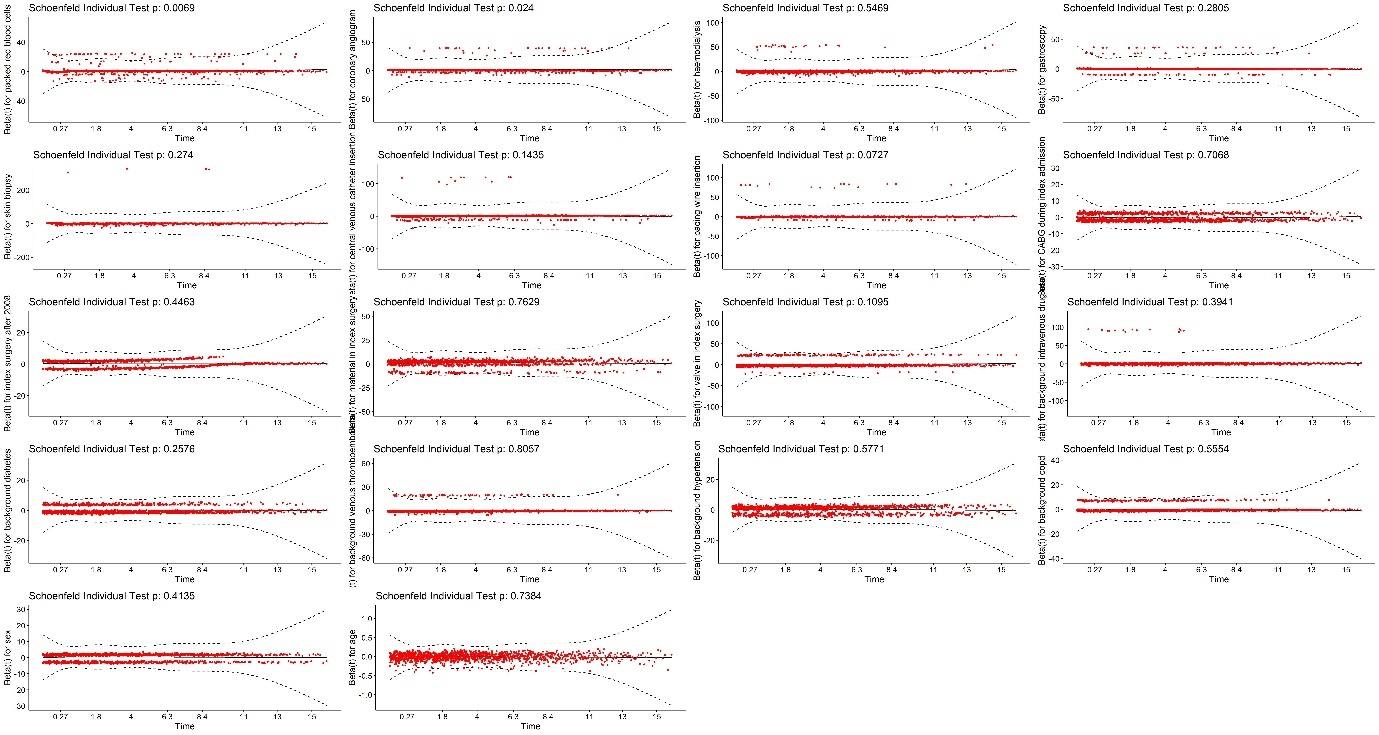


**Figure Legend**

Global p-value = 0.2597

**Figure S5: Schoenfeld residuals from multivariable regression model for risk of PVE (refer to Figure S3)**


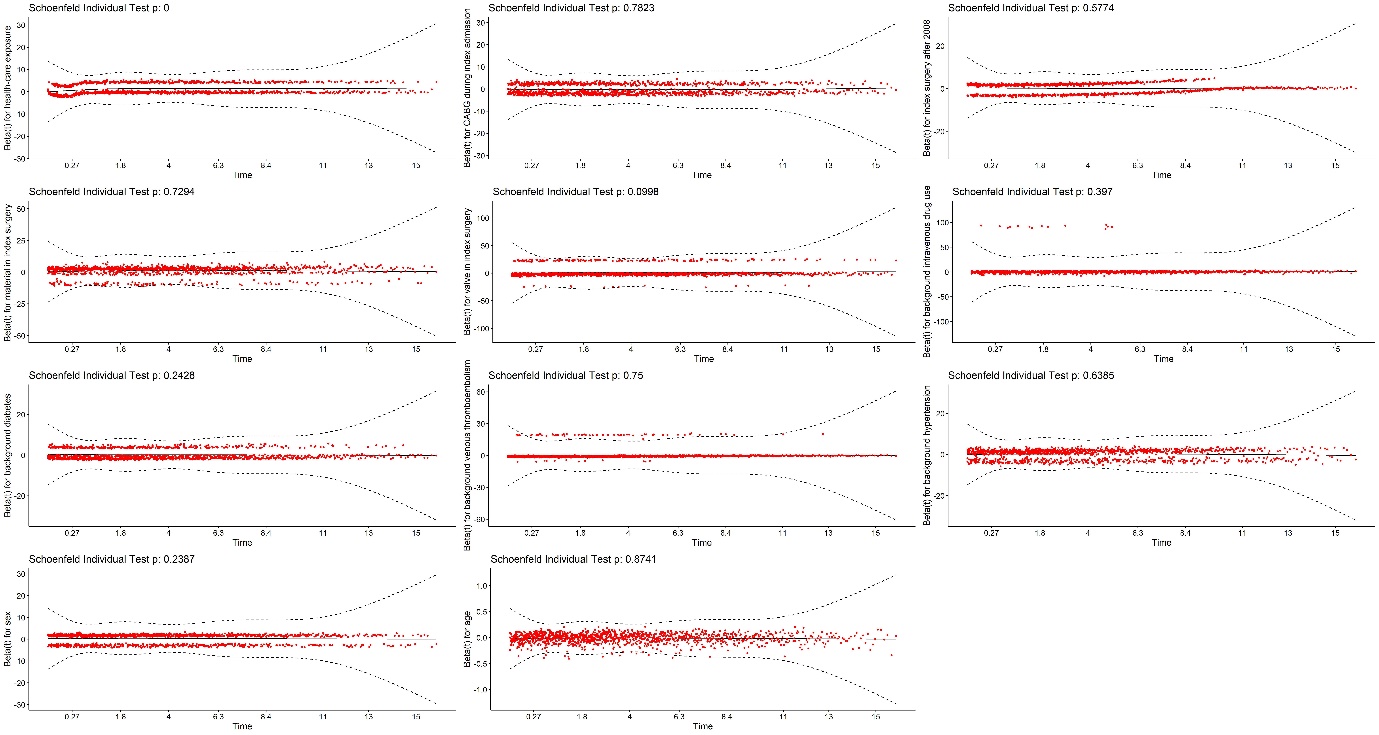


**Figure Legend**

Global p-value 0.010

**Figure S6: Schoenfeld residuals from multivariable regression model for risk of PVE (refer to Figure 4)**


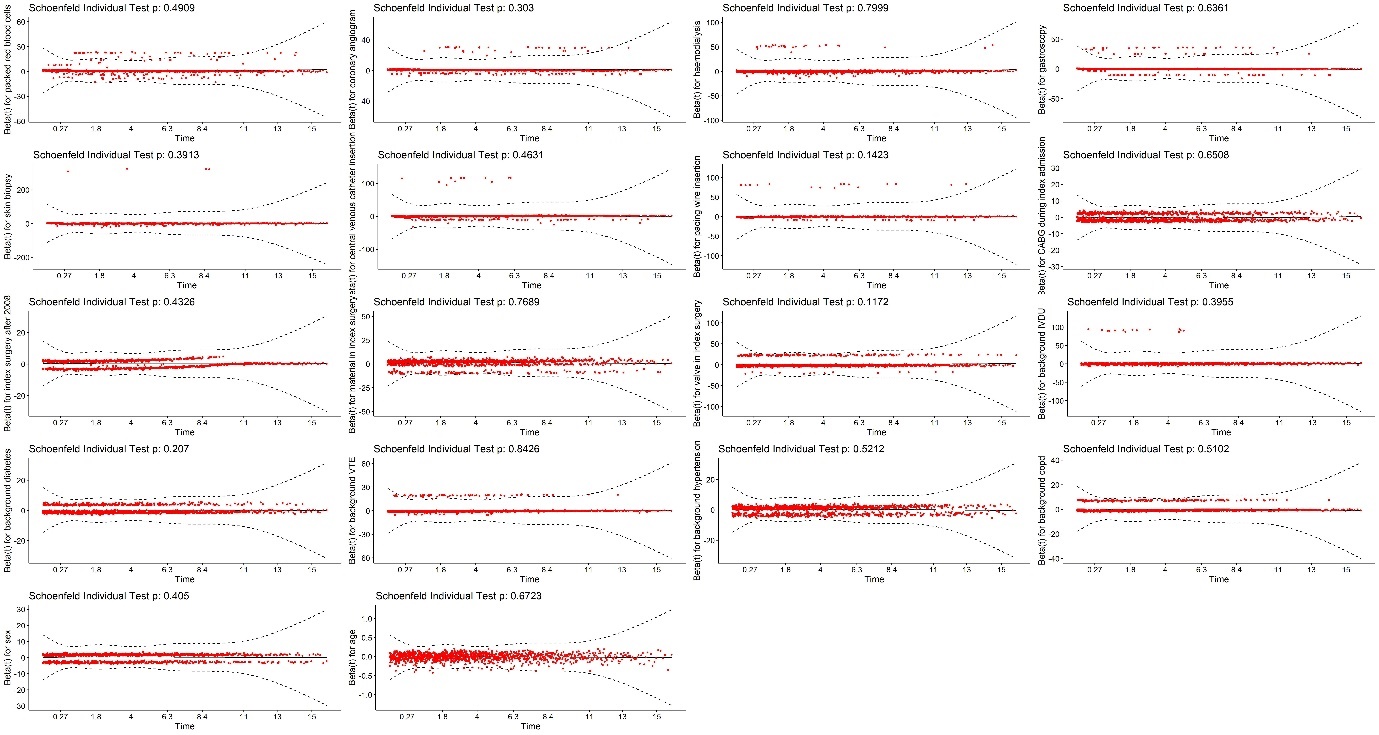


**Figure Legend**

Global p-value = 0.81
COPD = chronic obstructive pulmonary disease; IVDU = intravenous drug use; PVE = post cardiac valve surgery endocarditis; VTE = venous thromboembolism

**Figure S7: Schoenfeld residuals from multivariable regression model for risk of PVE (refer to Figure 5)**


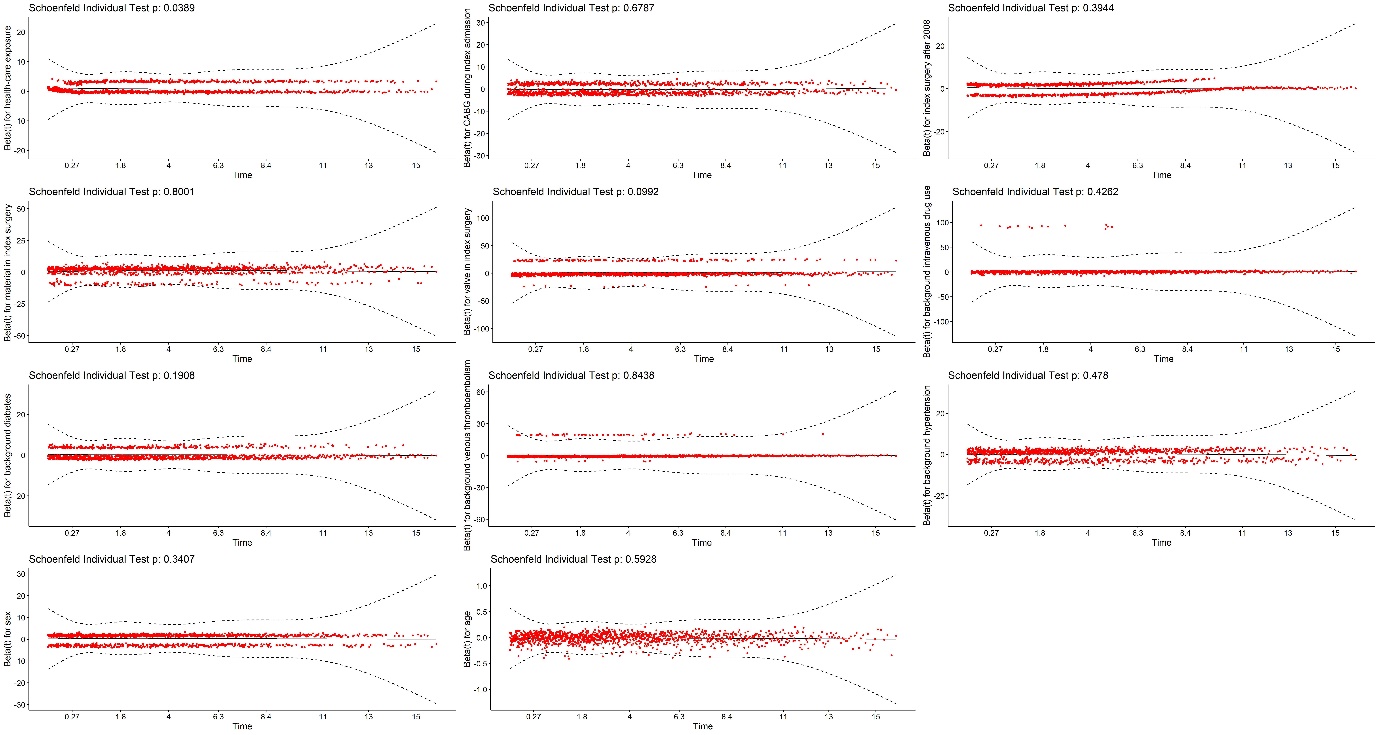


**Figure Legend**

Global p-value 0.227

**Figure S8: Incidence of healthcare exposures during 1 year prior to PVE in density plots**

| **(a)**  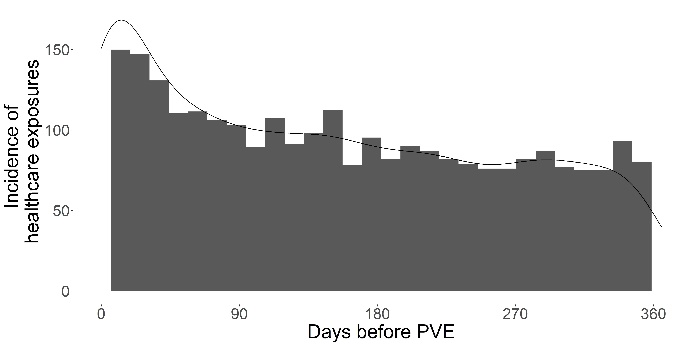 | **(b)**  **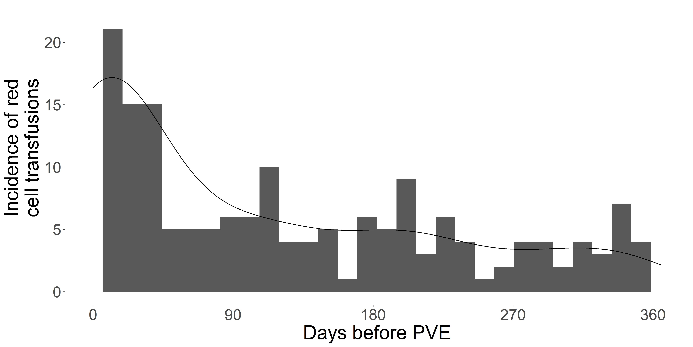** |
| --- | --- |
| **(c)**  **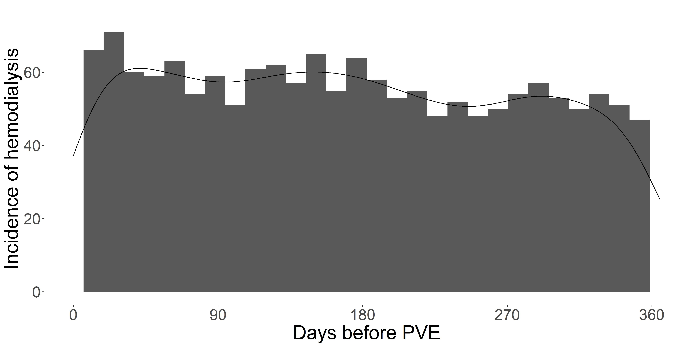** | **(d)**  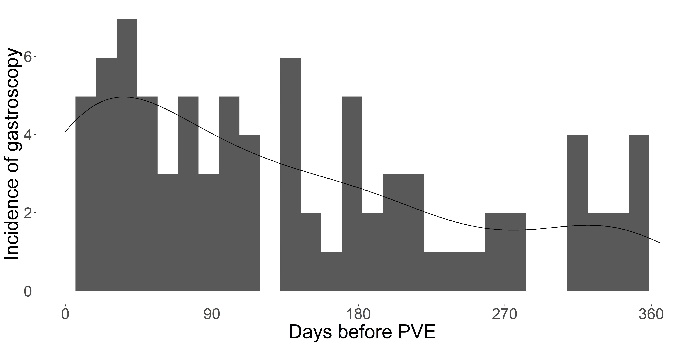 |
| **(e)**  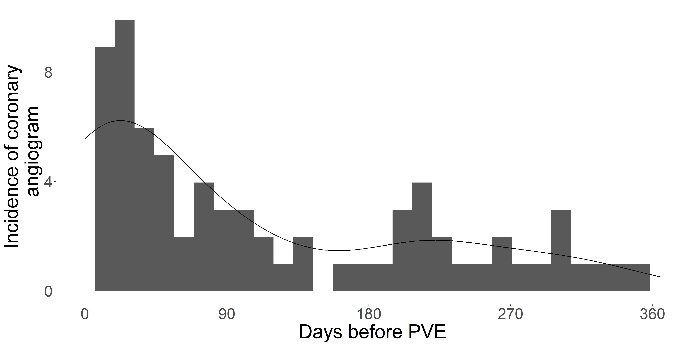 | **(f)** 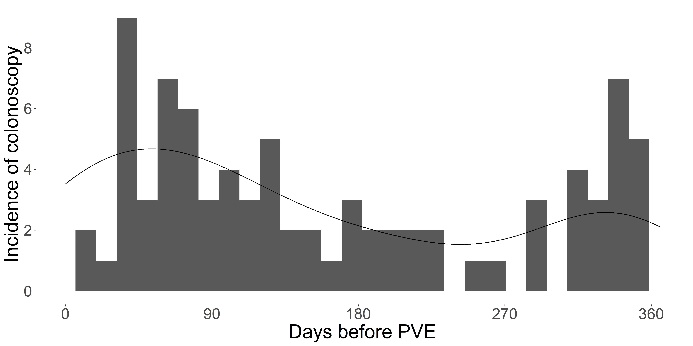 |

**Figure Legend**

Incidence shown of:
(a) any healthcare exposure
(b) red cell transfusion
(c) hemodialysis
(d) gastroscopy
(e) coronary angiogram
(f) colonoscopy

PVE = post cardiac valve cardiac surgery endocarditis
